# DNA methylation status at diagnosis in MDS predicts response to azacitidine

**DOI:** 10.1172/JCI208317

**Published:** 2026-09-29

**Authors:** Qin Yang, Miguel Torres-Martin, Masataka Taguchi, Alice Brogi, Irene Casalin, Eleonora Ceneri, Stephanie Halene, Amy E. DeZern, Elizabeth A. Griffiths, Matilde Y. Follo, Carlo Finelli, Jerald P. Radich, Michael J. Rauh, Rafael Bejar, Mikkael A. Sekeres, Valeria Santini, Maria E. Figueroa

**Affiliations:** 1 Department of Biochemistry & Molecular Biology, and; 2 Sylvester Comprehensive Cancer Center, University of Miami Miller School of Medicine, Miami, Florida, USA.; 3 MDS Unit, Hematology, DMSC University of Florence, AOU Careggi, Florence, Italy.; 4 Department of Biomedical and Neuromotor Sciences, University of Bologna, Bologna, Italy.; 5 Department of Internal Medicine, Yale University School of Medicine, New Haven, Connecticut, USA.; 6 Sidney Kimmel Comprehensive Cancer Center, Johns Hopkins Medicine, Baltimore, Maryland, USA.; 7 Roswell Park Comprehensive Cancer Center, Buffalo, New York, USA.; 8 Department of Experimental, Diagnostic and Specialty Medicine, Institute of Hematology L. and A. Seràgnoli, University of Bologna, S. Orsola-Malpighi Hospital, Bologna, Italy.; 9 Translational Science & Therapeutics Division, Fred Hutchinson Cancer Center, Seattle, Washington, USA.; 10 Department of Pathology and Molecular Medicine, Queen’s University, Kingston, Ontario, Canada.; 11 Division of Hematology and Oncology, UCSD Moores Cancer Center, La Jolla, California, USA.; 12 Division of Hematology and; 13 Department of Human Genetics, University of Miami Miller School of Medicine, Miami, Florida, USA.

**Keywords:** Genetics, Hematology, Epigenetics

## Abstract

**BACKGROUND:**

Myelodysplastic syndromes (MDS) are characterized by aberrant DNA methylation, and mutations in epigenetic modifiers are frequently found in these patients. Although DNA methyltransferase inhibitors are used to treat MDS, response variability remains a challenge in the clinic, with limited predictive markers.

**METHODS:**

We integrated genomic, epigenomic, and transcriptomic analyses of *n* = 98 patients with MDS. Patients were classified into epigenetic subtypes via hierarchical clustering. Random forest classifiers were developed and validated using internal stratified testing and an independent external cohort to predict azacitidine (AZA) response.

**RESULTS:**

MDS are characterized by widespread DNA hypomethylation affecting distal regulatory elements. We identified 7 epigenetic clusters correlated with distinct molecular drivers. Notably, cluster VI exhibited low mutational burden but a high AZA response rate of 71% (*P* ≤ 0.01). While transcriptional profiles alone failed to distinguish responders, a DNA methylation–based (DNAme-based) classifier achieved an area under the curve (AUC) of 0.82. An integrative model combining DNAme, gene expression, mutations, and clinical parameters achieved an AUC of 0.93 in internal validation and 0.88 in the external cohort.

**CONCLUSION:**

Epigenetic signatures at distal genomic elements provide superior predictive power for AZA response compared with promoter-centric or transcriptional analyses. These findings establish a robust framework for personalized treatment strategies in MDS.

**TRIAL REGISTRATION:**

ClinicalTrials.gov Identifier: NCT01522976.

**FUNDING:**

NIH National Heart, Lung, and Blood Institute grant: R01HL126947; Leukemia and Lymphoma Society: grants 13-5719, 7028-22.

## Abstract 2

Artificial intelligence tools help predict outcomes to treatments for MDS patients

## Graphical abstract

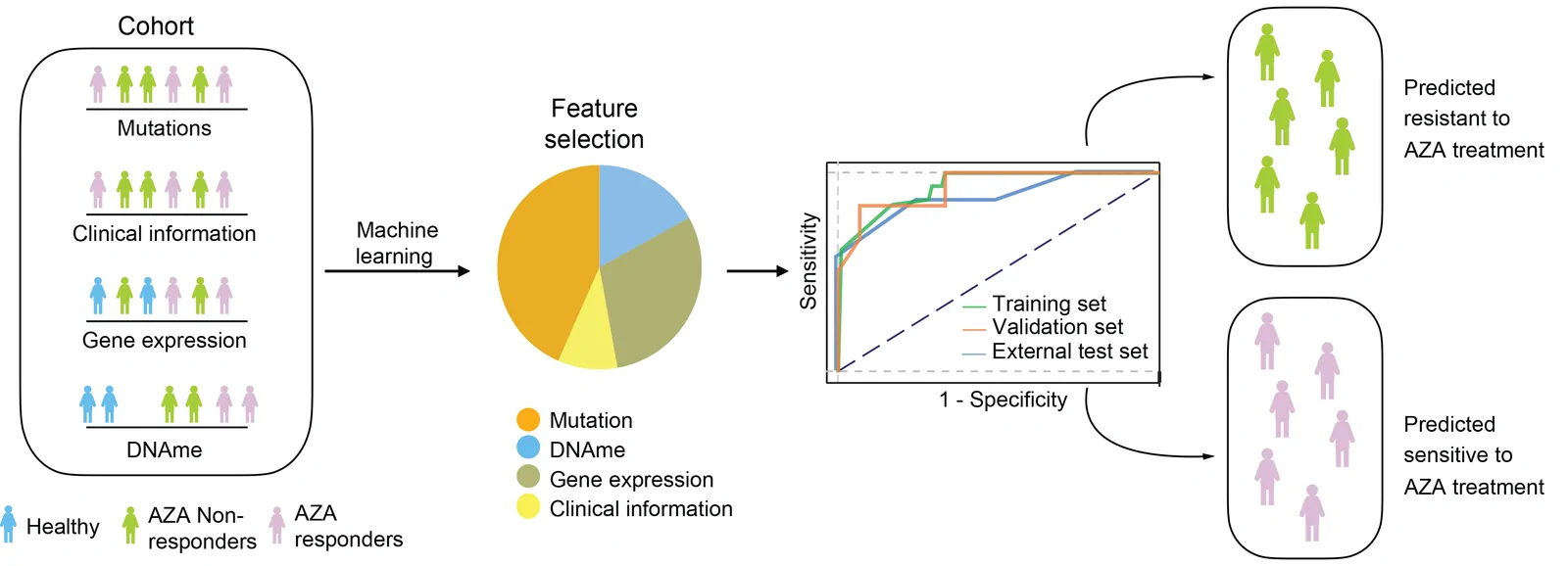

## Introduction

Myelodysplastic syndromes (MDS) encompass a heterogeneous group of disorders characterized by ineffective hematopoiesis, dysplastic changes in the bone marrow (BM), and the potential to progress to acute myeloid leukemia (AML) (1–3). Aberrant DNA methylation (DNAme) is a hallmark of MDS; however, its role remains incompletely understood. Early reports focused on aberrant hypermethylation of tumor suppressor gene promoters, e.g., p15 and E-cadherin (4–6), while the advent of DNAme microarrays revealed widespread aberrant hypermethylation at diagnosis (7), which directly correlated with disease aggressiveness and progression (8). Later molecular studies reported the existence of recurrent mutations in genes involved in epigenetic regulation, such as *TET2*, *ASXL1*, *DNMT3A*, and *EZH2*, in MDS (9–11). However, these groundbreaking early studies used smaller cohorts from single centers, no CD34^+^ selection, and analysis restricted to promoter methylation and gene expression microarrays.

Unlike de novo AML, MDS is more likely to be resistant to conventional chemotherapy (12, 13). For high-risk patients with MDS, while hematopoietic cell transplantation (HCT) offers the only curative option, it is usually available to only a small fraction of this older patient population. The remaining high-risk patients rely on treatment with DNA methyltransferase inhibitors (DNMTi) — azacitidine (AZA) or decitabine — the only available treatment option that has demonstrated a survival advantage compared with conventional care (14–18). However, the overall response rate to these agents is between 30% and 40%, responses may take longer than 6 months (17), and the duration lasts a median of 17 months. Consequently, for the majority of patients in real-world settings, these agents are ineffective, expose them to side effects, and result in significant time and economic burden (14). Once DNMTi have failed, survival in the absence of an allogeneic HCT is generally less than 6 months (19). It is thus critical to identify patients with MDS who are unlikely to benefit from DNMTi to accelerate access to HCT, offer up-front investigational studies, and, for frailer patients, offer the option of supportive care alone.

Despite some studies suggesting associations between the methylation reversal at select loci and clinical response to DNMTi (20–23), overall, earlier epigenetic studies have failed to identify robust associations between specific baseline DNAme profiles and DNMTi response in MDS (20, 22, 24, 25). More recently, Thoms and colleagues reported baseline and dynamic differences in DNAme in patients sensitive to AZA compared with nonresponders in the context of a phase II multicenter study. However, this study included chronic myelomonocytic leukemia (CMML) and patients with low-blast AML in addition to MDS, and the small size of the cohort precluded the development of robust epigenetic response classifiers (26). The absence of response classifiers impedes the customization of treatment plans for individual patients with MDS. To address this critical gap, we conducted a multicenter study of a cohort of AZA-treated patients with MDS. Through genomic, epigenomic, and transcriptomic analyses on CD34^+^ BM cells, we identified widespread aberrant DNAme, targeting distal regulatory elements at key biological pathways. These aberrant epigenomic patterns can be used to classify MDS into distinct epigenetic subtypes linked to specific genetic, molecular, and clinical characteristics. Furthermore, we harnessed these epigenetic profiles to develop robust, epigenetic-based classifiers that correlate with accurate pretreatment prediction of therapeutic response to AZA. These classifiers offer the potential to improve personalized treatment strategies and enhance outcomes for patients with MDS.

## Results

### MDS displays extensive aberrant DNA hypomethylation affecting distal regulatory elements.

To assess epigenetic deregulation in MDS, we analyzed pretreatment CD34^+^ BM specimens from a multicenter cohort of 98 International Prognostic Scoring System (IPSS) intermediate- or high-risk patients with MDS (27). The cohort consisted of cases from the North American Intergroup MDS trial S1117 (*n* = 37 cases), augmented with cases from US (*n* = 24) and Italian (*n* = 37) academic centers. All patients received AZA-based regimens and had documented responses. Patients were studied with targeted gene mutation profiling of 112 genes frequently mutated in MDS (*n* = 92 patients), DNAme by enhanced reduced representation bisulfite sequencing (ERRBS; *n* = 90 cases), and gene expression by RNA-seq (*n* = 77 cases) (Table 1 and Supplemental Table 1; supplemental material available online with this article; https://doi.org/10.1172/JCI208317DS1). We first compared gene expression profiles between CD34^+^ hematopoietic stem and progenitor cells (HSPCs) from patients with MDS and age-matched healthy individuals (*n* = 11). Unsupervised analysis using principal components analysis (PCA) revealed modest discrimination of the 2 groups (Figure 1A). This was confirmed by orthogonal approaches using hierarchical clustering and correspondence analysis (Supplemental Figure 1A). Furthermore, a direct comparison identified only 54 differentially expressed genes (DEGs; absolute fold-change [FC] ≥ 1.5 and a false discovery rate [FDR] ≤ 0.05; Supplemental Table 2) between MDS and controls (Supplemental Figure 1B). Comparison of this transcriptional profile with those from previous MDS cohort studies showed a high degree of consistency (Supplemental Figure 1C) (28–30). Gene set enrichment analysis (GSEA) identified upregulation of pathways related to interferon responses, IL6 signaling, oxidative phosphorylation, reactive oxygen species, cell cycle, and mitophagy in MDS (Supplemental Figure 1D and Supplemental Table 3).

Next, we investigated DNAme patterns in the same cohort of patients with MDS and samples from 10 healthy individuals (31) using ERRBS (median coverage: 2.6 million CpGs at ≥10× to ≤400× depth; Supplemental Table 4). PCA based on DNAme profiles demonstrated a clear separation between healthy individuals and patients with MDS (Figure 1A). Notably, unlike prior studies focusing solely on promoter regions that captured only aberrant DNA hypermethylation in MDS (7, 8), supervised analysis comparing the 2 groups identified significant hypomethylation (*n* = 4,196 hypomethylated and *n* = 3,785 hypermethylated differentially methylated regions [DMRs]; absolute methylation difference ≤ 25% and FDR ≤ 0.05) (Figure 1, B and C, and Supplemental Table 5).

Since ERRBS targets not only promoters but also intronic, exonic, and distal intergenic regions, we were able to explore the impact of aberrant hypo- and hypermethylation within specific genomic regions. Overall, DMRs were enriched at intergenic regions (DMRs 40% vs. background 33%, binomial test *P* value ≤ 0.001) while being less likely to target promoter regions (DMRs 16% vs. 25%, *P* value ≤ 0.001). Hypermethylated DMRs were depleted from intronic regions (hyper-DMRs 29% vs. 32%, *P* value ≤0.001), whereas hypomethylated DMRs displayed the opposite trend (hypo-DMRs 39% vs. 32%, *P* value ≤ 0.001; Figure 1D). Furthermore, we observed a significant enrichment of hypermethylated DMRs at CpG islands (hyper-DMRs 40% vs. 30%, *P* value ≤ 0.001) while hypomethylated DMRs were enriched at CpG island shores (hypo-DMRs 29% vs. 15%, *P* value ≤ 0.001; Figure 1D). When DMRs were annotated to hematopoietic enhancers (32), hypermethylated DMRs were found to be significantly depleted compared with background (hyper-DMRs 1.7% vs. 2.8%, *P* value ≤ 0.001) while hypomethylated DMRs were significantly enriched (hypo-DMRs 4.5% vs. 2.8%, *P* value ≤ 0.001; Figure 1D). Pathway enrichment analysis of genes annotated to these DMRs showed enrichment in various cancer, inflammation, and signaling pathways known to play a role in hematopoiesis and malignant transformation, such as the Hippo (33, 34), RAP1 (35, 36), calcium (37, 38), and PI3K-Akt (39–41) signaling pathways (Figure 1, E and F, and Supplemental Table 3).

Taken together, these findings underscore the magnitude of aberrant gains and losses in DNAme in MDS beyond promoter regions, identifying crucial DNAme changes targeting distal regulatory elements. Furthermore, they provide insight into the potential functional implications of DNAme alterations in MDS, suggesting their involvement in critical signaling cascades and molecular processes known to regulate HSPC biology.

### DNAme profiles capture the underlying molecular and clinical heterogeneity of the disease.

We hypothesized that DNAme profiles might capture aspects of MDS heterogeneity that are not fully reflected by molecular and cytogenetic profiling alone. Using hierarchical clustering of ERRBS data, we grouped patients with MDS in our cohort into 7 clusters and explored their association with clinical features (age, sex), laboratory parameters (complete blood counts, blast percentages), molecular and cytogenetic information, WHO 2022 classification, and AZA response. Molecular or cytogenetic features were considered cluster associated when they showed statistically significant enrichment and were present in more than 40% of patients in a given cluster (Table 2).

As expected, mutations in splicing factor proteins and epigenetic modifiers were very frequently seen in the cohort, and most clusters included patients carrying some of these mutations. However, as we had previously observed in the context of AML (42), DNAme cluster identity correlated strongly with specific clinical and molecular features (Figure 2, A and B), suggesting that DNAme patterns may provide complementary information on MDS heterogeneity beyond that captured by existing molecular and cytogenetic frameworks. Cluster II (*n* = 13) was enriched for patients carrying ASXL1 (Fisher’s test *P* value ≤ 0.01), STAG2 (*P* value ≤ 0.05), and RUNX1 (*P* value ≤ 0.05) mutations. Cluster III (*n* = 6) was defined by the presence of U2AF1 (*P* value ≤ 0.01) and BCOR (*P* value ≤ 0.01) mutations. Cluster IV (*n* = 6) was enriched for the presence of SF3B1 mutations (*P* value ≤ 0.001) co-occurring with RUNX1 mutations in women (*P* value ≤ 0.05). In contrast, 5/6 patients with SF3B1 outside of cluster IV were male and did not cluster together. Notably, 10/19 patients in cluster V carried mutations in TP53 (*P* value ≤ 0.001). In addition, patients in this cluster were almost devoid of splicing factor mutations, with only a few positive cases, most of which carried U2AF1 mutations. Moreover, cluster V was strongly enriched in men, with 16/19 male patients (*P* value ≤ 0.05). Notably, the remaining 8 cases with TP53 mutations were scattered among the other clusters and were mainly female (5/8). Cluster VII was enriched in mutations in the DNAme machinery (8/14), most of them (6/8) affecting DNMT3A (*P* value ≤ 0.05) (Supplemental Table 6).

Two of the clusters identified could not be classified based on any specific mutation, but instead, patients in those clusters shared robust epigenetic profiles and clinical features. Patients in cluster I (*n* = 13) were characterized by older age (median 78 years old, range = 69–85 vs. median 68, range = 37–86; *P* value ≤ 0.05) and intermediate-risk cytogenetics (*P* value ≤ 0.05), the majority of whom (*n* = 10) did not respond to DNMTi treatment, though this did not achieve statistical significance.

Finally, unlike patients in cluster II, in which most patients carried 3 mutations or more, cluster VI was striking because of its much lower mutation frequency than other clusters (Figure 2C and Supplemental Table 6). However, despite this overall paucity of mutations, it was noteworthy that 5 out of 7 patients harboring DDX41 mutations in our cohort (4 germline and 1 somatic) were located in this cluster (*P* value ≤ 0.001). Despite this, DDX41 mutations were not considered cluster defining as they affected only 5/14 patients (36%) in the cluster. In addition to a low frequency of mutations, patients in cluster VI showed a higher response rate to AZA than patients in other clusters (defined as a response of hematologic improvement or better within 6 months of beginning treatment), with 71% (10 out of 14) responding to the drug (*P* value ≤ 0.01; Figure 2D). In line with this paucity of mutations, 7 out of 12 patients from our MDS cohort who fell under the “Very low,” “Low,” or “Moderate low” 2022 IPSS-M (43) risk categories were classified together in this cluster (*P* value ≤ 0.01).

Notably, and consistent with our PCA findings, a comparable hierarchical clustering analysis of gene expression data failed to classify patients into robust clusters that were correlated with molecular and clinical features (Supplemental Figure 2). Taken together, these findings highlight the robustness of DNAme in capturing the molecular and clinical heterogeneity of MDS.

### Cluster VI patients have mild epigenetic deregulation and shared clinical and laboratory features.

To further characterize the epigenetic deregulation of these clusters, we compared them with healthy controls. We first conducted a PCA to visualize the epigenetic distances between controls and MDS patient clusters (Figure 3A). Notably, cluster VI, which carried very few mutations and responded well to AZA treatment, was epigenetically closer to the healthy controls than any of the other clusters, indicating the DNAme profiles of these patients are less perturbed. Supervised analysis comparing each cluster with the controls revealed that cluster VI had fewer aberrant DMRs than the other clusters. While the other 6 clusters all displayed widespread epigenetic abnormalities that clearly distinguished them from controls (with total DMRs ranging from 12,000 to 45,000), cluster VI had only 1,669 hypomethylated DMRs and 1,141 hypermethylated DMRs (Figure 3B, Supplemental Figures 3 and 4, and Supplemental Table 7). Furthermore, cluster VI exhibited significantly higher hemoglobin (Hb) levels than the other clusters (mean: 10.9 g/dL; range: 7.7–13.7 g/dL; ANOVA *P* value ≤ 0.05), once again indicating that patients in this cluster display more benign features than the rest of the cohort. Notably, there were no significant differences in BM blast percentage, red blood cells, platelets, white blood cells, or absolute neutrophil counts (ANC) between cluster VI and the other clusters (Figure 3C). To validate that cluster VI biology was not solely driven by the presence of *DDX41* mutations, we compared the response rates to AZA of the 5 *DDX41*-mutant cases with those of the 9 wild-type cases and found no statistically significant difference between them (4/5 vs. 6/9, Fisher’s test *P* value = NS). Likewise, there were no significant differences in mutation burden (Supplemental Figure 5A), and unsupervised clustering of cluster VI patients failed to segregate *DDX41-*mutant from wild-type cases (Supplemental Figure 5B). Finally, although Hb levels were higher in the *DDX41*-mutant cases (Supplemental Figure 5C), this did not translate to differences in clinical response, thus confirming that DNAme identified a biologically distinct group of patients that correlates more with the mutational burden than the specific mutations identified.

To understand the distinct epigenetic deregulation associated with MDS patient cluster VI, we annotated the identified DMRs to neighboring coding and noncoding transcripts. Pathway analysis revealed they targeted genes involved in IL-2 and IL-6 signaling, as well as signaling- and cancer-related pathways (Figure 3, D and E, and Supplemental Table 8). Comparing the transcriptional profiles between controls and cluster VI patients, we identified 1,182 downregulated and 1,040 upregulated genes (Supplemental Table 9). GSEA revealed that while a few of the same inflammation-related pathways seen in the entire cohort of MDS cases were also upregulated in cluster VI, this cluster was characterized by a less inflammatory profile overall, showing instead distinct enrichment of pathways involved in metabolism (Figure 3F and Supplemental Table 8).

These findings demonstrate how epigenetic profiles reveal potentially novel biology in MDS. Namely, we identified a distinct subgroup of patients with MDS, who presented with very few mutations and were instead defined by a unique DNAme signature and shared clinical and laboratory features as well as upregulation of metabolism pathways.

### Sensitivity to AZA is associated with specific epigenetic profiles at diagnosis.

Using International Working Group (IWG) 2006 criteria (44), we classified patients as responders (if they achieved a hematologic improvement or partial or complete response within 6 months of treatment initiation) and nonresponders (those with stable or progressive disease). PCA based on gene expression profiles could not effectively differentiate between AZA responders and nonresponders (Figure 4A), while a supervised analysis approach identified only 25 DEGs between the 2 groups (Figure 4B and Supplemental Table 10). Notably, comparison of this transcriptional profile with profiles from previous studies showed a high degree of consistency (Supplemental Figure 6) (26, 45, 46). GSEA revealed distinct pathway enrichment, with sensitivity to DNMTi associated with an expression profile characterized by a lower degree of inflammation (Figure 4C and Supplemental Table 11). Unsupervised analysis of DNAme profiles also failed to robustly segregate overall AZA responders or even best responders (i.e., those achieving a complete response; CR) from nonresponders (Supplemental Figure 7, A and B). Notably, cluster VI responder and nonresponder patients were clearly segregated away from all other responder and nonresponder patients, underscoring their unique biology (Supplemental Figure 7C). Supervised analysis revealed marked DNAme differences associated with response to AZA, consisting of 1,505 hypomethylated and 1,067 hypermethylated DMRs (Figure 4D and Supplemental Table 12). These DMRs associated with response to AZA were enriched at intergenic regions (DMRs 48% vs. 36% *P* value ≤ 0.001) and strongly depleted from promoter regions (DMRs 5% vs. 21%, *P* value ≤ 0.001) and CpG islands (DMRs 7% vs. 25%, *P* value ≤ 0.001) (Supplemental Figure 7D).

To further refine the epigenetic profiles associated with sensitivity to AZA, we repeated the analysis, restricting the AZA responder group to those who achieved CR and comparing them with nonresponders. This analysis identified 1,198 hypomethylated and 608 hypermethylated DMRs (Figure 4E and Supplemental Table 13). This epigenetic profile partially overlapped with that of all nonresponders (Figure 4F). Once again, DMRs were enriched at intergenic regions (DMRs 47% vs. 35%, *P* value ≤ 0.001) and were strongly depleted from promoter regions (DMRs 6% vs. 22%, *P* value ≤ 0.001) and CpG islands (DMRs 9% vs. 26%, *P* value ≤ 0.001) (Supplemental Figure 7E). Pathway analysis of response-associated DMRs revealed enrichment for the TGF-β and cell adhesion pathways (Figure 4G and Supplemental Table 14), suggesting that these may play a central role in the response to AZA.

In summary, comprehensive DNAme analysis provided valuable insights into the response of patients with MDS to AZA treatment. Moreover, the genomic distribution pattern of these response-associated DMRs explains why promoter-centric analyses did not identify any correlation between DNAme profiles and response to DNMTi and underscores the biological importance of epigenetic deregulation at distal genomic elements.

### DNAme profiles can be harnessed for the robust prediction of therapeutic response.

Given the existence of baseline DNAme differences in responders and nonresponders, we hypothesized that these unique methylation profiles could be harnessed to predict which patients would be sensitive or resistant to AZA treatment. To test this, we first used stratified randomization to divide patients into a training and independent validation cohort while ensuring a balanced inclusion of responders and nonresponders in both groups. The training and independent validation cohorts consisted of 63 and 27 patients, respectively. Importantly, to ensure fully independent validation and avoid potential overfitting of the model, differential methylation analysis as well as all steps of feature section and model building were performed only within the training cohort after the random split. Percentage cytosine methylation at 154 DMRs identified between responders and nonresponders, using only this 63-patient training cohort, was used as input to a machine-learning approach with random forest (RF) to build a classifier (Supplemental Table 15). Five-fold cross-validation was performed, and input features were ranked and selected based on their importance. Ninety-eight selected features were used to train the final RF model with the best parameters, which was only then applied to the 27 patients set aside for the independent blinded test set. The reported area under the receiver operating characteristic curve (ROC-AUC) on the test set was used to evaluate the classifier’s performance (Figure 5A). This DNAme RF classifier demonstrated the ability to predict AZA response with an AUC of 0.82, a sensitivity of 0.7, and a specificity of 0.77 (Figure 5B).

Despite this robustness, we hypothesized that incorporating clinical, mutation, and gene expression information would further improve its performance. Since previous results showed only a few genes with differential expression between responders and nonresponders, we first sought to identify genes whose expression is likely epigenetically regulated by DNAme. We previously reported that analyzing gene expression–methylation correlations within functional genomic units determined by topologically associating domains (TADs) leads to the identification of genes directly or indirectly regulated via DNAme (47). Therefore, we used 2,711 TAD regions identified in healthy CD34^+^ HSPCs and, for each sample for which we had gene expression, mutation, clinical information, and DNAme data available (*n* = 69), we calculated the Pearson’s correlation between the DNAme level of all the DMRs between responders and nonresponders and the gene expression value of each gene transcript within the same TAD. As a result, 4,612 genes were found to be highly correlated with 1,092 DMRs within 820 TADs, with an absolute *r* ≥ 0.7. Next, we used these genes in a network analysis to identify relevant networks of epigenetically regulated genes. The top network identified consisted of 15 genes forming a highly interconnected network with CCL5 as the hub node (Figure 5C). Notably, several genes in this network were regulated via DMRs located at long distances from their loci, again underscoring the important role that distal events play on epigenetic deregulation in MDS (Supplemental Figure 8A).

Using this cohort of patients with available DNAme, RNA-seq, mutation, and clinical information, we randomly assigned patients to a training cohort (*n* = 48) and an independent validation cohort (*n* = 21). As in the first model, feature selection was performed exclusively using the training cohort to prevent information leakage. Due to differences in sample availability and cohort composition, DMRs were reidentified independently for this model, resulting in 192 DMRs. In addition to DNAme features, the expression from our CCL5 network (*n* = 25), mutation data (*n* = 69 genes), and clinical parameters (*n* = 7) previously shown to be relevant to MDS biology and prognosis (48–52) were incorporated (Supplemental Table 16). Following feature selection, 53 features were selected, consisting of a combination of clinical, mutation, expression, and DNAme data. Among the clinical data, Hb, age, platelet count, BM blast percentage, and ANC were included in the final model. Twenty-three gene mutations were included in the RF model. While some of them have been shown to have prognostic impact in MDS — e.g., NPM1 (53), TP53 (54), FLT3 (55, 56), and CUX1 (57, 58) — most of them have not been previously implicated as either prognostic or predictive of response to therapy in MDS. Notably, TET2 mutations, for which conflicting findings regarding their correlation with AZA response have been reported (59–61), had the lowest feature importance in our model, while ASXL1 and DNMT3A mutations, which have also been reported to correlate with response (62–64), were not selected at all. When applied to the 21 patients in the blinded independent test set, this new prediction model yielded an AUC of 0.93, with a sensitivity of 0.83 and a specificity of 0.93, demonstrating the increased robustness of this integrative classifier for predicting AZA response (Figure 5, D and E). To further assess the robustness of our classifier, we used an MDS cohort published by Thoms et al. (*n* = 11 cases) as an independent validation cohort (26). After retraining our model on the 45 overlapping features, the performance remained strong, yielding an AUC of 0.88 (Figure 5F).

To determine the relative importance of the different types of features included, we also tested predictive models, excluding each of the different data types one at a time. Exclusion of the DNAme data resulted in the classifier’s lowest performance, with an AUC score of 0.54 (Supplemental Figure 8B). Conversely, including DNAme while excluding gene expression features from the same model improved performance to an AUC score of 0.79, with a sensitivity of 0.8 and a specificity of 0.83. However, this was still significantly lower than the complete classifier’s performance (Supplemental Figure 8C). Exclusion of either mutation or clinical data also resulted in a decrease in the classifier’s performance, with AUC scores of 0.78 and 0.79, respectively (Supplemental Figure 8, D and E).

These findings indicate that DNAme data significantly contribute to the predictive power of classifier models, even to a greater extent than gene expression, mutation, and clinical and laboratory data. However, the highest performing model was achieved by integrating all 4 types of information, underscoring the synergism among the different features.

## Discussion

The slow DNMTi response and the necessity for ongoing treatment present significant challenges in assessing treatment effectiveness and adjusting therapeutic regimens promptly. By integrating genomic, epigenomic, and transcriptomic data, we gained insights into the complex biological networks underlying MDS heterogeneity, identifying those likely to respond to AZA. Understanding critical genomic regions, gene expression patterns, mutations, and clinical parameters related to AZA sensitivity is essential for developing more targeted and effective therapies. Our study offers initial insights into long-range epigenetic gene regulation mechanisms in MDS pathogenesis. We found that genomic regions affected by DNAme primarily reside in distal regulatory areas. Integrating Hi-C and Hi-ChIP 3D genome information from normal CD34^+^ cells revealed how DMRs control the expression of distal genes, confirming previous findings in AML (47) and emphasizing the need to focus beyond promoter regions to understand epigenetic dysregulation at distal regulatory elements in MDS. Future functional validation, such as using CRISPR-based epigenetic modulation in MDS cell models, will be essential to definitively establish the mechanistic impact of these distal regulatory elements on therapeutic response.

While we acknowledge the use of historical IWG 2006 and IPSS criteria, our findings remain clinically relevant as we have integrated contemporary frameworks, including the International Consensus Classification (65), the fifth edition of the WHO Classification (66), and the IPSS-M, for prognostication. Importantly, our study addresses a key gap in these current systems, which often overlook the epigenetic and transcriptomic dimensions essential for a comprehensive understanding of MDS heterogeneity. In this context, we utilized DNAme-based hierarchical clustering to assess whether epigenetic patterns capture additional dimensions of MDS heterogeneity beyond established molecular subtypes. Notably, this approach identified a subset of patients (cluster VI) that exhibited a distinct clinical and molecular profile, including few mutations, near-normal Hb, low-risk IPSS-M, and a higher proportion of responders to AZA treatment. Though it shares features with the CCUS-like group described by Bernard et al. (67), that group included both MDS and CMML cases, which are distinct disorders with different molecular profiles and therapy responses. Furthermore, DDX41 mutations, linked to AML and MDS predisposition and favorable outcomes (68), were more prevalent in this cluster, contrasting with the Bernard classification that separates patients with DDX41 mutation despite their shared features with others, as demonstrated in our study. We note that while our findings underscore the importance of DNAme in MDS biology, the relatively modest cohort size and the resulting small cluster sizes limit their generalizability. Therefore, these results should be interpreted as exploratory epigenetic associations that require further validation in larger and more diverse patient populations. Moreover, the presence of clonal hematopoiesis cannot be ruled out in the healthy controls utilized for comparison, as paired mutational data were not available. This limitation must be kept in mind when interpreting disease-specific epigenetic profiles identified in this study.

While prior studies have linked specific mutations (59, 60, 69–76) or expression profiles (28, 29, 45) to improved response rate to DNMTi, they did not consistently correlate with improved survival and were not reproducible across other studies. We report the development of robust predictive classifiers by integrating DNAme, gene expression, mutations, and clinical and laboratory parameters. This approach was critical in enhancing the predictive power of our model. Specifically, mutations in 23 genes — including *NPM1*, *RAD21*, and *TP53* — along with the expression patterns of 16 genes, such as *C5AR2*, *C3*, and *TNFRSF9*, substantially contributed to the model’s performance. While some of these mutations and gene expression patterns have been reported in previous studies on myeloid disorders (77–80), they have not been collectively used as biomarkers for AZA response. Notably, excluding DNAme from the model resulted in a significant decline in performance, highlighting the importance of DNAme information in capturing disease biology and improving prediction accuracy. Our findings highlight the potential of epigenetic data as predictive biomarkers and emphasize the importance of integrating multiple molecular features for improved predictive accuracy. While this classifier was robust in predicting response in an independent validation cohort, we recognize that our findings represent an exploratory biomarker study. Prospective validation in larger cohorts will be necessary to fully validate these classifiers and ensure their generalizability before they can be considered for incorporation into routine clinical practice.

In conclusion, our integrative approach sheds light on the complexity of MDS and underscores how this molecular interplay influences treatment response. This holistic perspective can offer valuable insights into the molecular mechanisms underlying AZA response in MDS and may ultimately lead to more effective and personalized treatment strategies. Further exploration of the roles of biological pathways identified will be critical for advancing our understanding of MDS pathogenesis and therapeutic approaches. Furthermore, the integration of emerging single-cell multiomic technologies will likely provide even higher resolution into the cellular heterogeneity of the MDS hematopoietic stem and progenitor cell compartment, potentially identifying rare cell populations or subclonal epigenetic states that drive therapeutic resistance.

## Methods

### Sex as a biological variable.

Both men and women were included in this study. Sex distribution was comparable between responders and nonresponders and was assessed using Fisher’s exact test. As no significant association between sex and response status was observed, men and women were analyzed together. The results are therefore expected to be applicable to both sexes.

### MDS samples.

Clinical BM specimens were collected from 98 deidentified MDS specimens classified as intermediate- or high-risk MDS by the IPSS (27). Mononuclear cells collected from BM aspirates at the time of diagnosis were isolated through Ficoll density centrifugation and frozen for later use.

CD34^+^ cells were isolated using MACS magnetic beads (Miltenyi Biotec). DNA and RNA were extracted with the QIAGEN AllPrep kit. Further details are provided in the Supplemental Methods.

### Statistics.

All statistical analyses were performed using R statistical software (version 4.1, R-Core Team, http://www.R-project.org/). For sequencing-based assays, significance was defined as FDR ≤ 0.05; for clinical comparisons, *P* ≤ 0.05 was used. Additional statistical details are provided in the Supplemental Methods. For box plots, data are shown as median with 25th–75th percentile ranges; whiskers extend to minimum and maximum values; and individual outliers are represented by dots.

### Study approval.

Institutional Review Board (IRB) approval was obtained at the University of Miami Miller School of Medicine, Yale University School of Medicine, Roswell Park Cancer Institute, University of Florence, University of Bologna, and the Southwestern Oncology Group central IRB and local IRBs of participating institutions. Written informed consent was obtained from all patients at the time of collection in accordance with the Declaration of Helsinki.

### Data availability.

The ERRBS and bulk RNA-seq data have been deposited in the National Center for Biotechnology Information’s Gene Expression Omnibus database under the accession code GSE281562 (RNA-seq) and GSE281610 (ERRBS). Additional ERRBS data for healthy donors were obtained from GSE52945, samples GSM1536373, GSM1536374, and GSM1536375. For RNA-seq data from healthy donors, 5 of them were from a previous publication (81). Code used for the analyses in this study is available on GitHub: https://github.com/santur90/MDS_AZA; commit ID: c8d6b1c. Values for all data points in graphs are reported in the Supporting Data Values file.

## Manuscript information

### Author contributions.

MEF conceived and designed the study and supervised all aspects of the analysis and data interpretation. QY and MTM analyzed genome-wide data. RB analyzed mutational data. MT contributed to clinical data organization and annotation. AB, IC, EC, SH, EAG, MYF, CF, JPR, MJR, MAS, AED, and VS contributed samples. MAS and VS provided critical feedback and contributed to data interpretation. QY and MEF wrote the manuscript with approval from all coauthors.

### AI disclosure.

Microsoft 365 Copilot (Premium; Microsoft Corporation) was used from April through July 2026 to assist with grammar correction and language editing during manuscript drafting and revision.

### Conflict of interest.

RB reports employment and equity from Aptose Biosciences; consulting fees from Bristol-Myers Squibb (BMS), Servier, Geron, Ipsen, Keros, Gilead, BigSur; and scientific advisory board membership for NeoGenomics. EAG reports advisory board/honoraria fees from AbbVie, Alexion Pharmaceuticals/AstraZeneca Rare Disease, Apellis Pharmaceuticals, Celgene/BMS, CTI Biopharma, Genentech, Novartis, Picnic Health, Servier, Takeda Oncology, and Taiho Oncology and research funding from Astex Pharmaceuticals, AstraZeneca Rare Disease/Alexion Pharmaceuticals, Apellis Pharmaceuticals, Blueprint Medicines, Genentech, and NextCure. MAS serves on the advisory board for BMS. AED reports consulting fees from BMS, Novartis, Takeda, Pfizer, and Agios.

### Funding.

This study was supported by the following:

- NIH National Heart, Lung, and Blood Institute R01HL126947 to MEF.
- Leukemia and Lymphoma Society (LLS) Scholar Award (13-5719) to MEF.
- LLS SCOR (7028-22) to MEF.
- Onco-Genomics Shared Resource of the Sylvester Comprehensive Cancer Center at the University of Miami, by the National Cancer Institute (NCI) of the NIH under award number P30CA240139.
- Roswell Park Comprehensive Cancer Centers Biorepository and Laboratory Services Shared Resource, by NIH NCI grant P30CA016056.
- Associazione Italiana per la Ricerca sul Cancro (AIRC) IG-26537-2021 Investigator Research Grant to VS.

This work is the result of NIH funding, in whole or in part, and is subject to the NIH Public Access Policy. By accepting this federal funding, the NIH has the right to make the work publicly available in PubMed Central.

### Present addresses.

MTM’s present address is: Centre for Genomic Regulation, Barcelona Institute of Science and Technology, Barcelona, Spain. MT’s present address is: Department of Hematology, Nagasaki University, Nagasaki, Japan.

## Supplementary Material

Supplemental data

ICMJE disclosure forms

Supplemental table 1

Supplemental table 2

Supplemental table 3

Supplemental table 4

Supplemental table 5

Supplemental table 6

Supplemental table 7

Supplemental table 8

Supplemental table 9

Supplemental table 10

Supplemental table 11

Supplemental table 12

Supplemental table 13

Supplemental table 14

Supplemental table 15

Supplemental table 16

Supporting data values

## Figures and Tables

**Figure 1:**
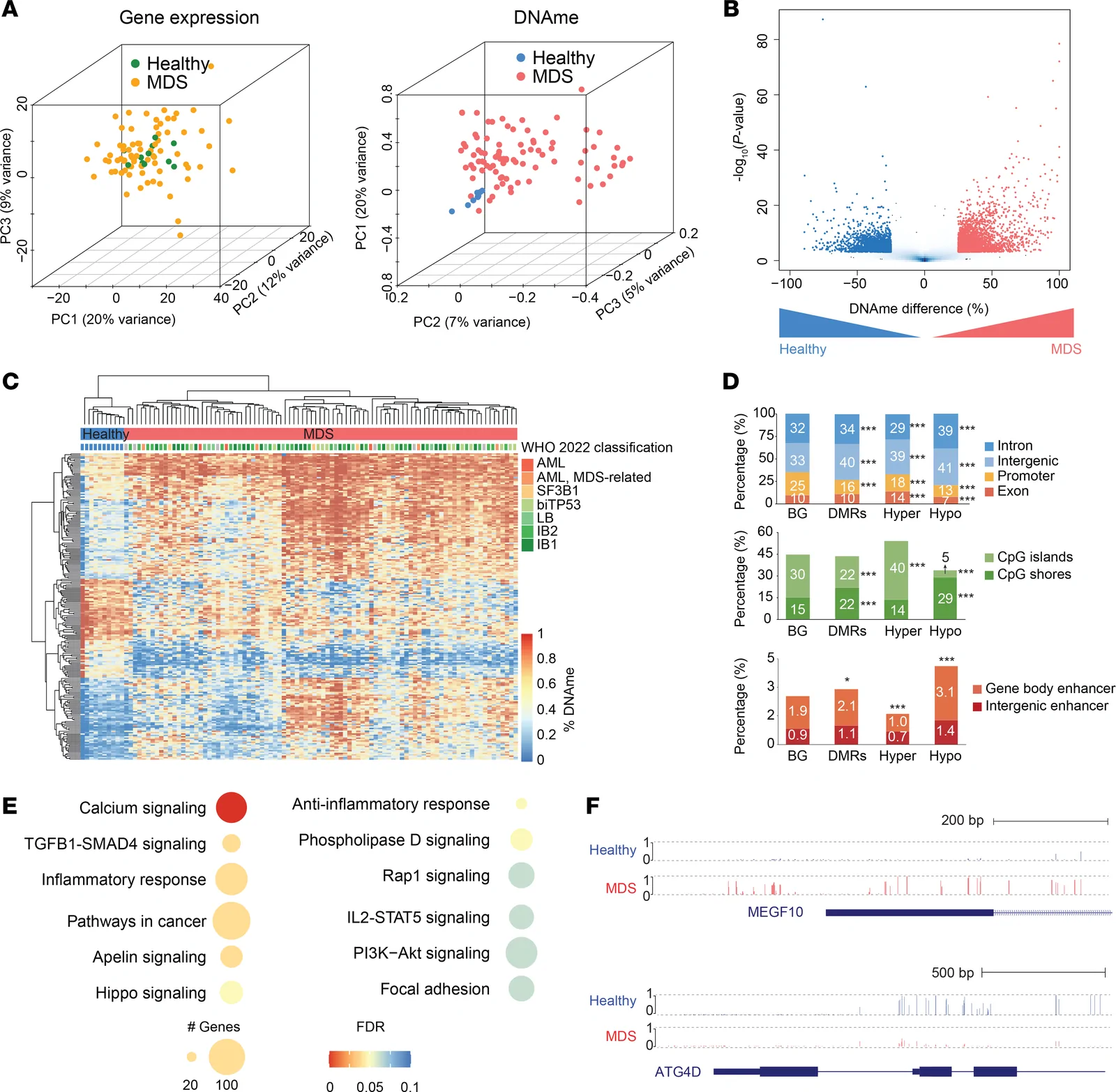
Comprehensive analysis of DNAme alterations in MDS. (**A**) Principal components analysis of RNA-seq data (left) from healthy controls (shown in green) and patients with MDS (shown in yellow), along with enhanced reduced representation bisulfite sequencing (right) data from healthy controls (blue) and patients with MDS (red). (**B**) Volcano plot depicting methylation differences versus the –log_10_(*P* value) between healthy controls and patients with MDS. Hypomethylated and hypermethylated regions in MDS are shown in blue and red, respectively (absolute methylation difference ≥ 25%, FDR ≤ 0.05). (**C**) Heatmap with hierarchical clustering illustrating methylation levels of differentially methylated regions (DMRs) (without missing values) in healthy controls (blue bar) and MDS patients (red bar), annotated with WHO 2022 subtypes. WHO 2022: IB1, MDS with increased blasts-1; IB2, MDS with increased blasts-2; LB, MDS with low blasts. (**D**) Stacked bar plot showing the distribution of DMRs across genomic regions, CpG islands, and enhancers relative to background (BG) distribution. *P* values were calculated using a binomial test. *, *P* ≤ 0.05; ***, *P* ≤ 0.001. (**E**) Bubble plot depicting enriched pathways (FDR ≤ 0.1) for MDS-associated DMRs, using all CpG tiles as the background. Bubble size corresponds to the number of genes in each pathway; color indicates statistical significance. (**F**) UCSC Genome Browser tracks of 2 representative loci illustrating hypermethylated (top) and hypomethylated (bottom) regions in MDS. Bar height represents the methylation level of each region.

**Figure 2:**
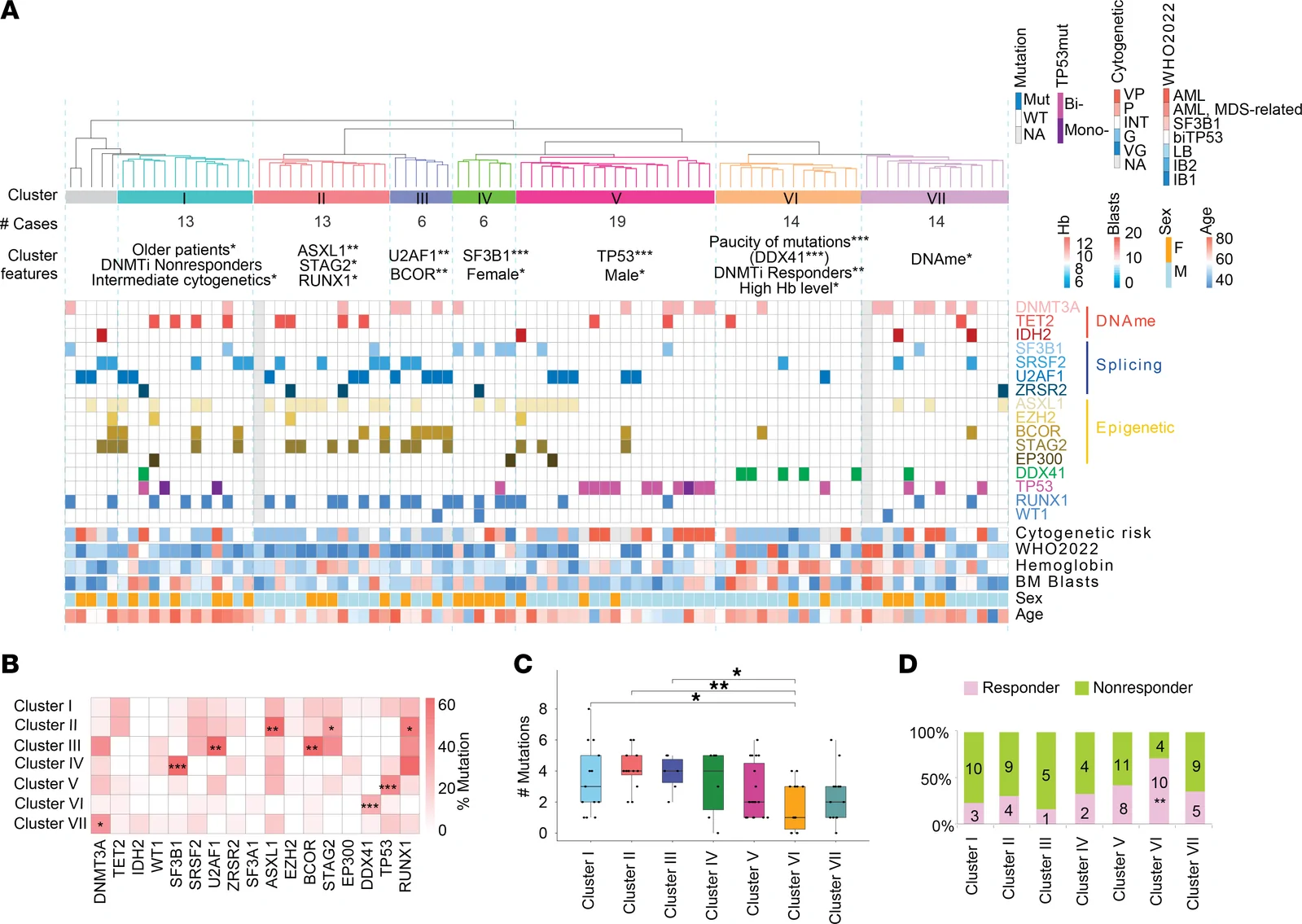
DNAme captures molecular heterogeneity in MDS. (**A**) Heatmap with hierarchical clustering illustrating the 7 MDS patient clusters identified based on DNAme data, with corresponding mutation and clinical annotations displayed below. Patients harboring gene mutations are color-coded. Cytogenetic risk: VP, very poor; P, poor; INT, intermediate; G, good; VG, very good; NA, not available. WHO 2022: IB1, MDS with increased blasts-1; IB2, MDS with increased blasts-2; LB, MDS with low blasts. *, *P* ≤ 0.05; **, *P* ≤ 0.01; ***, *P* ≤ 0.001. (**B**) Heatmap depicting the mutation profiles of each identified cluster. *P* values were calculated using Fisher’s exact test. *, *P* ≤ 0.05; **, *P* ≤ 0.01; ***, *P* ≤ 0.001. (**C**) Box plot showing the number of mutations per patient across the identified clusters. The bounds of the boxes represent the 25th and 75th percentiles, the horizontal line within the box indicates the median, and the whiskers extend to the minimum and maximum values. Individual outliers are represented by dots. *P* values were calculated using the Wilcoxon rank sum test. *, *P* ≤ 0.05; **, *P* ≤ 0.01. (**D**) Bar plot displaying the number of clinical responders and nonresponders within each cluster. *P* values were calculated using Fisher’s exact test. **, *P* ≤ 0.01.

**Figure 3:**
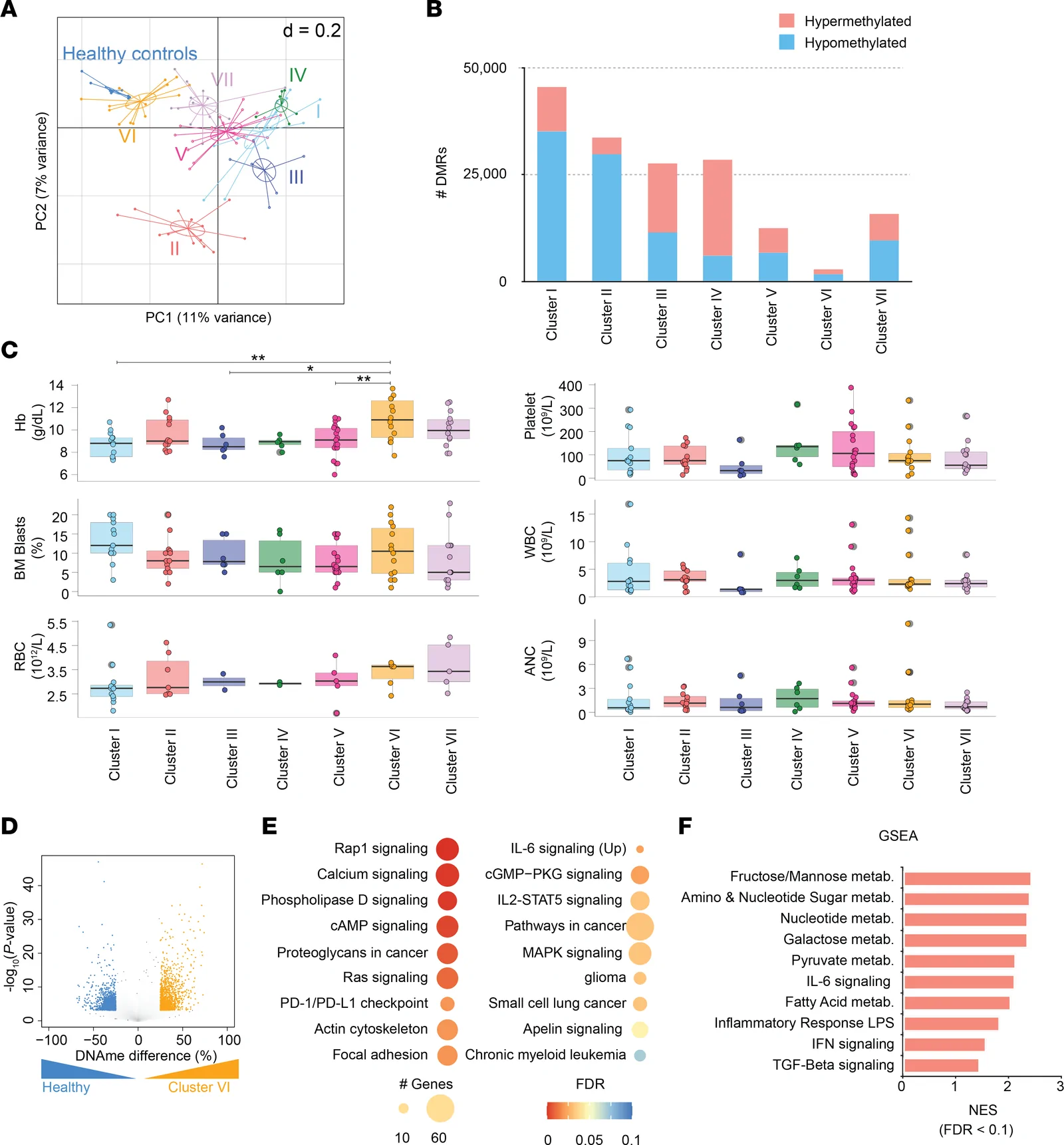
Epigenetic and clinical characteristics associated with each DNAme cluster. (**A**) Principal components analysis of healthy controls (blue) and MDS patient clusters. (**B**) Bar plot showing the number of hypermethylated (red) and hypomethylated (blue) regions in each MDS cluster compared with healthy controls. (**C**) Box plot illustrating the distribution of clinical features across MDS patient clusters. The bounds of the boxes represent the 25th and 75th percentiles, the horizontal line within the box indicates the median, and the whiskers extend to the minimum and maximum values. Individual outliers are represented by dots. *P* values were calculated using 1-way ANOVA with a post hoc Tukey’s honestly significant difference test. *, *P* ≤ 0.05; **, *P* ≤ 0.01. (**D**) Volcano plot illustrating methylation difference versus the –log_10_(*P* value) between healthy controls and MDS cluster VI. Significant hypomethylated (blue) and hypermethylated (orange) regions in MDS cluster VI are highlighted (absolute methylation difference ≥ 25%, FDR ≤ 0.05). (**E**) Bubble plot depicting enriched pathways (FDR ≤ 0.1) associated with DMRs in MDS cluster VI. Bubble size corresponds to the number of genes within each gene set, while color indicates statistical significance. (**F**) Bar plot showing the normalized enrichment score (NES) for pathways enriched in MDS cluster VI compared with healthy controls. Pathways with increased activity are shown in red.

**Figure 4:**
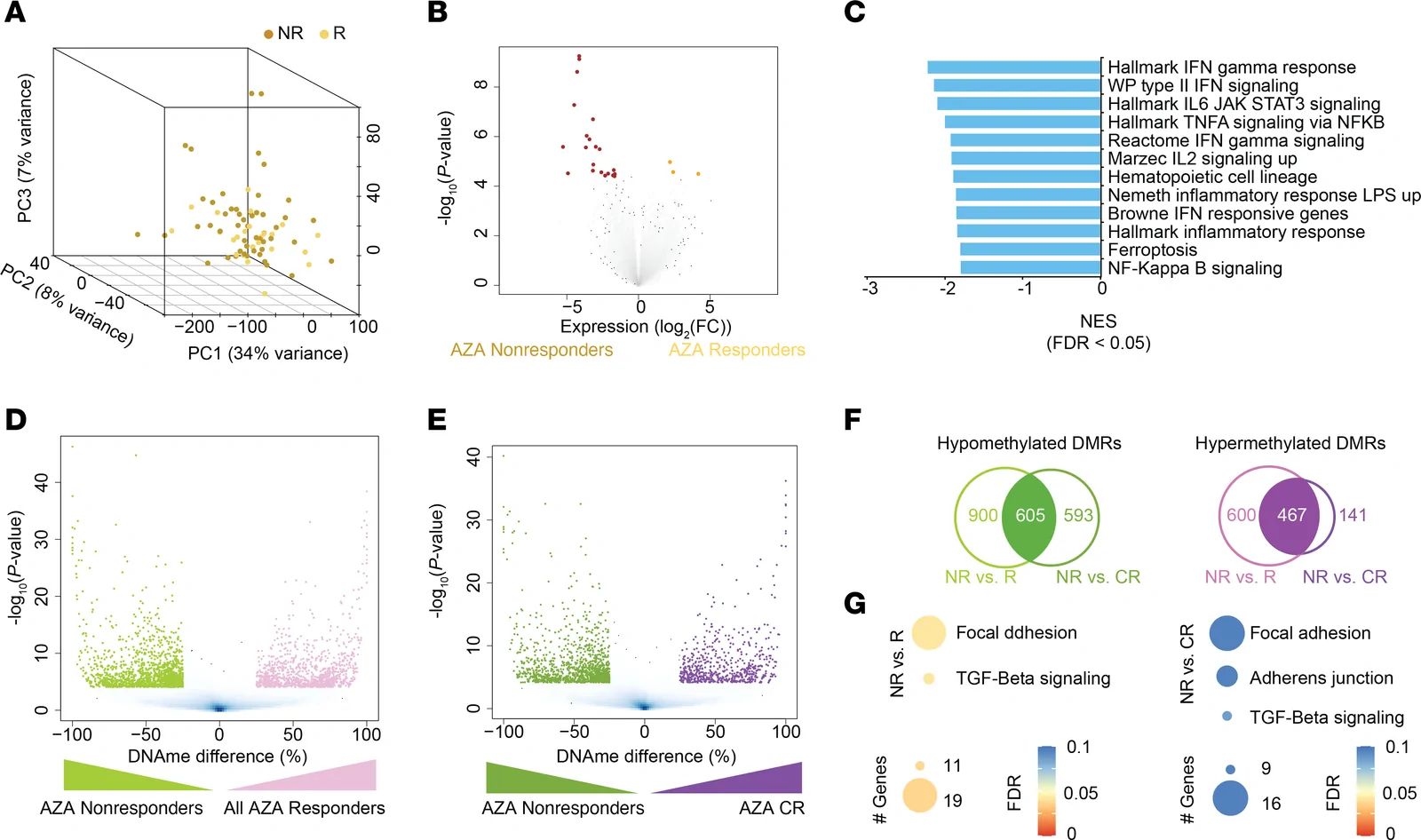
Epigenetic differences distinguish AZA responders and nonresponders before treatment. (**A**) Principal components analysis of RNA-seq data from AZA nonresponders (brown) and responders (yellow). (**B**) Volcano plot of the log_2_fold-change (AZA responders/nonresponders) gene expression versus the –log_10_(*P* value). Significantly downregulated and upregulated genes in AZA responders are shown in brown and orange, respectively (absolute fold-change ≥ 1.5, FDR ≤ 0.05). (**C**) Bar plot displaying the NES for pathways differentially regulated in AZA responders versus AZA nonresponders. Downregulated pathways are shown in blue (FDR ≤ 0.05). (**D**) Volcano plot showing methylation difference in methylated regions versus the –log_10_(*P* value) between AZA responders and AZA nonresponders. Significantly hypomethylated and hypermethylated regions in AZA responders are colored in green and light purple, respectively (absolute methylation difference ≥ 25%, FDR ≤ 0.05). (**E**) Volcano plot depicting methylation difference versus the –log_10_(*P* value) between AZA complete responders and AZA nonresponders. Significantly hypomethylated (green) and hypermethylated (dark purple) regions in AZA complete responders are highlighted (absolute methylation difference ≥ 25% and FDR ≤ 0.05). (**F**) Venn diagrams illustrating the overlap of DMRs between AZA nonresponders vs. responders and AZA nonresponders vs. complete responders. (**G**) Bubble plot showing enriched pathways (FDR ≤ 0.1) associated with hypomethylated and hypermethylated regions in AZA responders versus AZA nonresponders (left), as well as AZA complete responders versus nonresponders (right). Bubble size represents the number of genes within each gene set; color indicates statistical significance.

**Figure 5:**
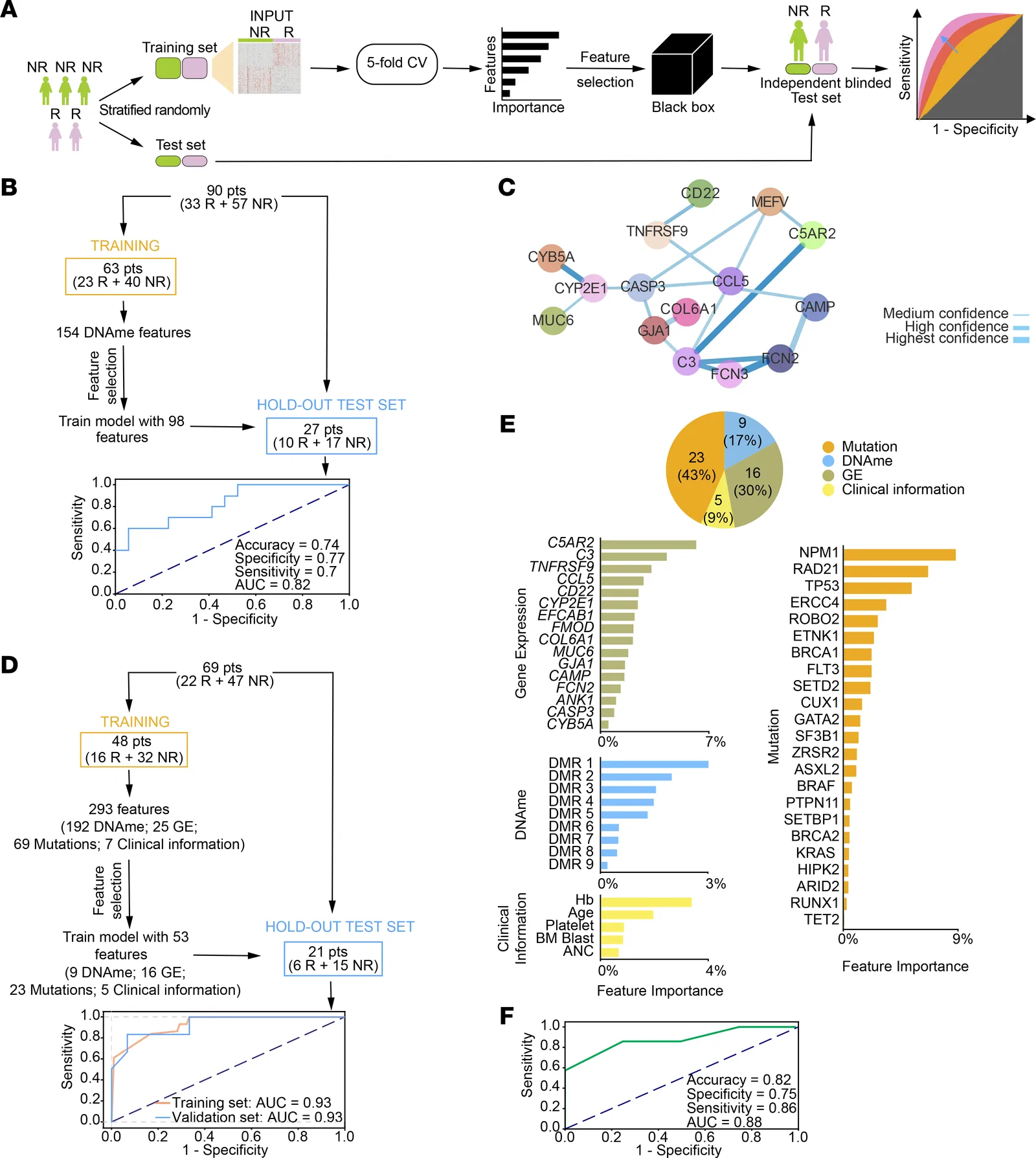
Machine learning models predict response to AZA at baseline. (**A**) Schematic representation of the machine learning model used to identify predictive features for AZA response at diagnosis. CV, cross validation (**B**) Workflow of machine learning predictor utilizing DNAme features to distinguish AZA responders from nonresponders. The ROC curve of biomarker performance is shown. (**C**) Protein-protein interaction network of genes associated with DMRs within the same topologically associating domain. Line thickness represents connection confidence levels. (**D**) Workflow of an integrated machine learning predictor incorporating DNAme, gene expression, mutations, and clinical features. The ROC curve of biomarker performance is shown. (**E**) Pie chart illustrating the distribution of selected features from the model in **D** (top) and bar plot depicting the importance of each selected feature (bottom). (**F**) ROC curve showing the performance of prediction on the external validation set.

**Table 2:**
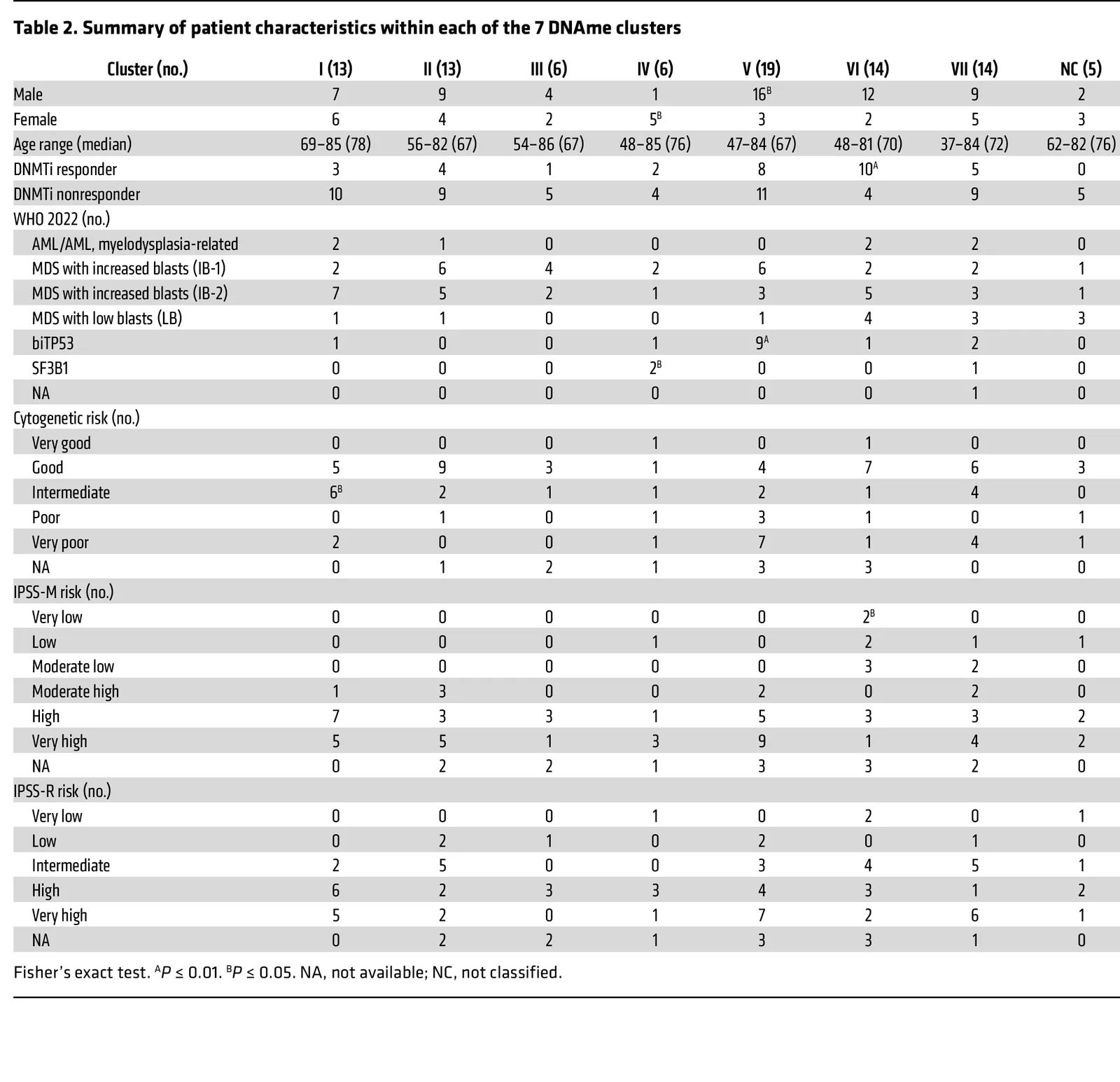
Summary of patient characteristics within each of the 7 DNAme clusters

**Table 1:**
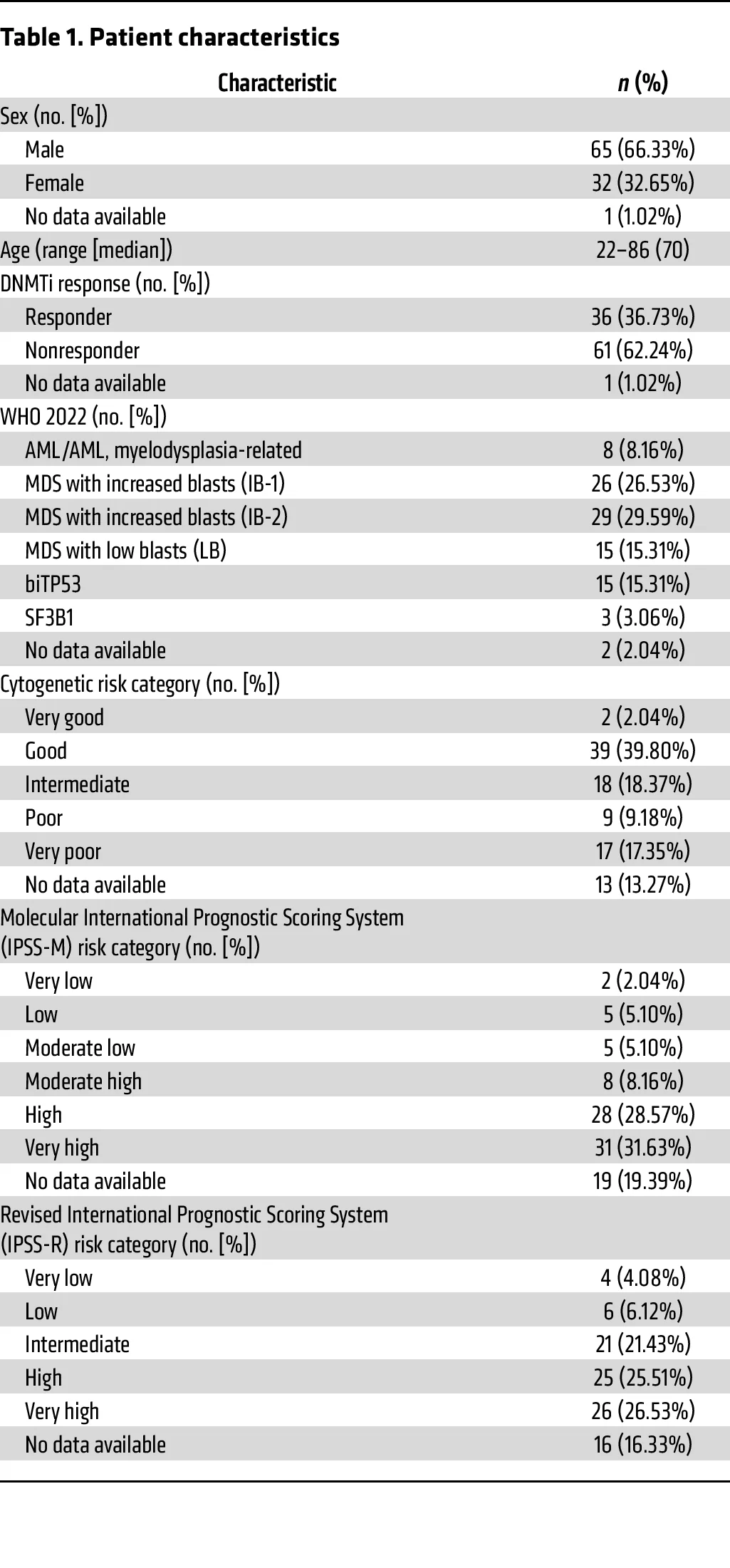
Patient characteristics

## Version 1 09/29/2026

In-Press Preview

## Version 2 11/16/2026

Electronic publication
