# Supplementary material for: DNA methylation status at diagnosis in MDS predicts response to azacitidine
Source: J Clin Invest. 2026 Sep 29;136(22):e208317. doi: 10.1172/JCI208317 (PMC13767612; doi:10.1172/JCI208317)
Supplement: Supplementary file 1 [file jci-136-208317-s166.pdf]

Supplementary material for

Unique epigenomic signatures identify biologically significant subtypes of MDS and predict response to azacitidine

Qin Yang[1,2], Miguel Torres-Martin[2,§], Masataka Taguchi[1,2,$], Alice Brogi[3], Irene Casalin[4], Eleonora Ceneri[4], Stephanie Halene[5], Amy E. DeZern[6], Elizabeth A. Griffiths[7], Matilde Y. Follo[4], Carlo Finelli[8], Jerald P. Radich[9], Michael J. Rauh[10], Rafael Bejar[11], Mikkael A. Sekeres[2,12*], Valeria Santini[3*], Maria E. Figueroa[1,2,12,13*#]

1. Dept. of Biochemistry & Molecular Biology, University of Miami Miller School of Medicine, Miami, FL, USA
2. Sylvester Comprehensive Cancer Center, University of Miami Miller School of Medicine, Miami, FL, USA
3. MDS Unit, Hematology, DMSC University of Florence, AOU Careggi, Florence, Italy
4. Dept. of Biomedical and Neuromotor Sciences, University of Bologna, Bologna, Italy
5. Dept. of Internal Medicine, Yale University School of Medicine, New Haven, CT, USA
6. Sidney Kimmel Comprehensive Cancer Center, Johns Hopkins Medicine, Baltimore, MD, United States
7. Roswell Park Comprehensive Cancer Center, Buffalo, NY, USA
8. Dept. of Experimental, Diagnostic and Specialty Medicine, Institute of Hematology L. and A. Seràgnoli, University of Bologna, S. Orsola-Malpighi Hospital, Bologna, Italy
9. Translational Science & Therapeutics Division, Fred Hutchinson Cancer Center, Seattle, WA, USA
10. Department of Pathology and Molecular Medicine, Queen's University, Kingston, Canada
11. Division of Hematology and Oncology, University of California San Diego Moores Cancer Center, La Jolla, CA, USA
12. Div. of Hematology, University of Miami Miller School of Medicine, Miami, FL, USA
13. Dept. of Human Genetics, University of Miami Mil;ljler School of Medicine, Miami, FL, USA

*contributed equally
[§]currently at: Centre for Genomic Regulation, Barcelona Institute of Science and Technology, Barcelona, Spain
[$]currently at: Dept. of Hematology, Nagasaki University, Nagasaki, Japan

[#]Corresponding author:
Maria E. Figueroa, MD
Professor,
Dept. of Biochemistry & Molecular Biology
Sylvester Comprehensive Cancer Center
1501 NW 10th Ave, BRB 709A
Miami, FL 33136
Email: mefigueroa@med.miami.edu
Phone: 305-243-7333

SUPPLEMENTARY Methods:

Human bone marrow samples:

CD34+ cells were isolated from MNC specimens using the Miltenyi MACS magnetic bead purification kit. Frozen human mononuclear cells (MNC) vials were thawed at 37°C, followed by treatment with 200 μL of DNase-I (Roche) at a concentration of 1 mg/mL for 90 seconds. Subsequently, the cells were gently resuspended in Iscove's Modified Dulbecco's Medium (IMDM) containing 40% fetal bovine serum (FBS) to minimize primary human cell mortality. All centrifugation steps were performed at 1500 rpm. After an 8-minute centrifugation, the cells were resuspended in 15 mL of MACS buffer and then incubated with magnetic beads for 50 minutes at 4°C. Following three consecutive washes with MACS buffer (each for 10 minutes at 4°C), the cells were resuspended in 6 mL of MACS buffer and passed through an AUTOMACS instrument using the POSSELD program. After another 8-minute centrifugation at 4°C, the cells were resuspended in RTL buffer with beta-mercaptoethanol. DNA and RNA were extracted from the CD34+ fraction using the AllPrep Micro kit (QIAGEN), while the CD34+ depleted fraction was processed with the AllPrep Mini kit.

Mutational sequencing:

Genomic DNA was employed to create libraries for hybrid capture following the manufacturer's protocol from Agilent. Libraries were assessed and then combined in equimolar amounts, aggregating to 500 mg of DNA for up to 24 samples per reaction.

The hybridization process was executed using Agilent Custom SureSelect In Solution Hybrid Capture RNA baits, designed to target 443 kbp of exonic DNA with the aid of 16,890 probes, as detailed in the work of Lindsley and colleagues (1). Each capture reaction underwent a sequence of washing, amplification, and was sequenced on two lanes of an Illumina HiSeq 2000, generating 100-bp paired-end reads.

Variant Calling

The analysis proceeded as follows: Fastq files were aligned to the human genome version hg19 using The Burrows Wheeler Aligner (BWA v0.7.12) MEM module, tailored for paired-end reads (2). To eliminate redundancy, duplicate reads were identified and removed through Picard tools (V1.91). For variant calling, GATK v3.2 was employed after performing base recalibration. Local realignments for insertions and deletions (indels) were executed using reference variant databases (3). Somatic variants were identified with LoFreq v2.1.1, considering all variants with a frequency of at least 1% (4). Further filtering of variants occurred post-variant calling, involving specific parameters: variant frequency below 0.05, read depth at the variant site less than 20, GQ and/or QUAL scores less than 30, and IndelRepeatFilter exceeding 8. Variants exhibiting excessive strand bias and indels with Variant Allele Frequency (VAF) less than 10% adjacent to homopolymer repeats were excluded through manual curation. Filtered variants were initially annotated using ANNOVAR (5). Variants that were predicted to modify splicing were evaluated as described in the work of Jian and colleagues (6). Variants located outside the protein-coding regions or splice sites of the genes listed earlier and synonymous variants not predicted to impact splicing were excluded.

To eliminate common polymorphisms, variants with population frequencies equal to or greater than 1% in either the 1,000 Genomes (7) or the Exome Aggregation Consortium (ExAC v.3.1) datasets were likewise removed, unless they were also documented as confirmed somatic mutations in the Catalogue of Somatic Mutations in Cancer (COSMIC) (8). Remaining variants underwent manual review, and those deemed probable somatic-coding variants were included in subsequent analyses. It's worth noting that the variant calling and interpretation processes were conducted without knowledge of sample identifiers and any associated phenotype information.

Enhanced reduced representation bisulfite sequencing:

ERRBS was carried out according to established protocols (9), with adjustments made to suit the low input of DNA. Before adapter ligation, the methylated adapters were diluted to a concentration of 150 nmol/L. During the gel size selection step, fragments falling within the range of 150 to 450 base pairs were excised. Subsequently, the prepared libraries were subjected to sequencing on an Illumina HiSeq 3000 platform.

Unsupervised clustering analyses (ERRBS):

DNA methylation data were first filtered to retain 25-bp CpG tiles with high variability across samples (top 30% by standard deviation). Pairwise sample similarity was then assessed using Kendall rank correlation, a non-parametric measure robust to outliers. A correlation-based dissimilarity matrix (1 − Kendall correlation) was used as input for hierarchical clustering, which was performed using Ward's linkage to generate compact

and biologically interpretable clusters. Cluster assignments were determined by applying a cutoff to the resulting dendrogram based on tree height.

Unsupervised clustering analyses (RNA-seq):

RNA-seq expression data were filtered to retain genes with high variability across samples (top 30% by standard deviation). Principal component analysis (PCA) was performed using the prcomp function in R (v4.1) (10), and the proportion of variance explained by each principal component was extracted from the PCA output. Correspondence analysis (COA) was conducted using the ord function from the ade4 package (v1.7.22) (11), and the proportion of inertia explained by each COA axis was calculated based on the corresponding eigenvalues. Unsupervised hierarchical clustering was performed using the same approach as described for the ERRBS data.

Differentially methylated regions analysis:

Reads were trimmed using Trim Galore (version 0.6.2) (12) and aligned to a bisulfite-converted human genome (hg19) using Bowtie2 (13) and Bismark (14) (versions 2.4.1 and 0.23.1, respectively). After strand collapsing and filtering by coverage (keep loci with reads between 10-400), methylation loci were tiled by window (25bp) and a methylDiff object was created using MethylSig (15) (version 1.5.0) for regions that were present in at least 3 samples per group. Significant differentially methylated regions (DMRs) were identified as those with an absolute mean methylation difference of ≥ 25 and a DMR q-value of ≤ 0.05. DMRs were annotated to the hg19 reference genome using RefSeq gene annotations (16). DMRs overlapping annotated gene features were classified as promoter,

exon, or intron regions. DMRs not overlapping any annotated gene features were classified as intergenic. Enhancer regions were defined using enhancer-promoter interaction maps derived from H3K27Ac HiChIP data in CD34+ HSPCs (17), and DMRs overlapping these enhancer regions were annotated as enhancer-associated DMRs. For gene assignment, DMRs overlapping a gene body or promoter region were assigned to their corresponding genes. The remaining intergenic DMRs were assigned to target genes based on corresponding enhancer-promoter interactions (17). We then performed functional annotation with all called methylation tiles as background using ChiPseeker (18) version 1.28.3. Heatmaps were generated with pheatmap (19) 1.0.12, where percent methylation of each DMR for each sample was calculated and plotted using the complete clustering method with Euclidian distance. NA values were depicted in light grey.

RNA sequencing

RNA extracted from the CD34+ fraction was employed for RNA-seq. RNA quality was evaluated using an Agilent Tapestation to guarantee high RNA quality. The RNA was treated with Illumina Ribo-Zero. For library preparation, we used the Nugen Ovation Solo RNA-seq ultra-low input kit, following the manufacturer's instructions. Subsequently, the prepared libraries were sequenced on an Illumina NextSeq platform. These libraries were paired-end and they were strand-specific. To normalize the data across runs, ERCC spike-ins were introduced.

RNA-seq alignment:

The quality of the resulting reads was assessed using FastQC (20) (v0.11.8) and multiQC (21) (1.11). Then all reads were processed using Trim Galore (12) (version 0.6.2) to trim to paired end 50 basepairs and remove adapters. The resulting reads were aligned to the hg19 Gencode v19 reference genome using the STAR (22) aligner (version 2.7.6a) with specific parameters: outFilterType=BySJout, outFilterMultimapNmax=20, alignSJoverhangMin=8, alignSJDBoverhangMin=1, outFilterMismatchNmax=999, alignIntronMin=20, alignIntronMax=1000000, alignMatesGapMax=1000000, and alignEndsType=EndToEnd.

Differential gene expression analysis:

Gene expression was quantified using featureCounts (23) (v2.0.1), which was run in paired-end mode with the hg19 gencode annotation file (excluding entries for ribosomal RNA). Differential expression analysis was performed using DESeq2 (24) (v1.32.0) with a multifactor design that accounted for both the donor's condition and any batch effects. Dispersion was calculated across both groups, and pairwise contrasts were established. Genes with an absolute fold change of at least 1.5 and a p-value adjusted for multiple comparisons of less than or equal to 0.05 were considered significant.

MDS Classification:

The MDS patient cohort was annotated using the International Prognostic Scoring System (IPSS) (25), the Molecular International Prognostic Scoring System (26), the Revised IPSS (IPSS-R) (27), and the 5th edition of the Classification (28). For example, under WHO 2022 criteria, MDS-bi *TP53* includes cases with either two or more *TP53* mutations,

or one *TP53* mutation accompanied by a complex karyotype involving the chromosome on which *TP53* is located.

GSEA:

Using a pre-ranked list of genes based on the Wald statistic from the DESeq2 (24) output, Gene Set Enrichment Analysis (GSEA) (29) (v4.2.3) was conducted. The gene set size was limited to 15-500 genes, and a weighted enrichment score was utilized.

DNA methylation to gene expression correlation analysis:

Hi-C libraries from healthy CD34+ HSPCs were sequenced as paired-end 150 bp reads with two biological replicates, yielding approximately 8×10^8 read pairs per replicate. Raw sequencing reads were processed using the Juicer pipeline (v1.5.6) (30). Briefly, reads were aligned to the hg19 reference genome using BWA, chimeric reads were identified, and duplicate read pairs were removed to retain valid unique contacts. For domain-level analysis, topologically associating domains were identified using the Arrowhead algorithm at 40-kb resolution for each replicate independently. 2,711 TADs showing overlap between replicates were merged to generate a consensus TAD set, which was used for downstream analyses (31). Differentially methylated regions that exhibited coverage between 10-400 in more than 5 samples for both AZA responders and non-responders were chosen for conducting Pearson correlation analysis. The correlation analysis involved examining the transcripts per million (TPM) values of all expressed genes within the same TAD region as the selected DMRs. Highly correlated pairs of DMRs and genes with absolute correlation coefficient ≥ 0.7 were identified.

Regulatory network identification:

Genes highly correlated with DMRs were analyzed using the STRING (32) database (v11.5) to construct protein-protein interaction networks. Then, network topology measures such as degree centrality and betweenness centrality were calculated using the NetworkAnalyzer plugin in Cytoscape (33) (v3.9.1) to identify highly connected and influential nodes in the network. The top sub-networks with a confidence score of at least 0.4 (medium confidence) were extracted and visualized using Cytoscape. The nodes were colored and made transparent based on their bundling strength, and the thickness of the edges represented the confidence of the correlations between genes. The identified nodes were further analyzed for their functional annotations and pathways using Kyoto Encyclopedia of Genes and Genomes (KEGG) (34) pathway enrichment analyses.

Machine learning classifier:

The samples were randomly stratified and divided into training and testing cohorts, with one-third of the samples reserved for testing purposes using train_test_split() function from sklearn (35) (v.1.2.2), ensuring balanced representation of responders and non-responders. The DNA methylation levels (without missing values) and/or gene expression levels, mutation status, and other clinical parameters were extracted for both the training and testing cohorts. The resulting matrices were standardized using the StandardScaler() function to ensure comparability of means and standard deviations. To maintain independence between training and validation, all feature selection steps (including identification of DMRs and differentially expressed genes) were performed exclusively

within the training cohort for each model. For the first model (DNAme only), 25-bp methylation tiles were generated as described above, and regions present in at least three samples per condition were retained using MethylSig. DMRs were selected based on the following criteria: absolute mean methylation difference ≥ 15, DMR q-value ≤ 0.1, and complete data coverage across all samples in the cohort (i.e no missing values were allowed). This resulted in 154 DMRs used for model training. For the second model (integrative model), the same pipeline was applied to a smaller cohort with available ERRBS, RNA-seq and mutation data across all samples. A stricter threshold of absolute mean methylation difference ≥ 20 was used to limit feature dimensionality that resulted in the identification of 192 DMRs.

For feature selection, Recursive feature elimination, L1-based feature selection and mutual information based techniques were employed. GridSearchCV() with 5-fold cross validation was performed to optimize model hyperparameters.

With the optimal parameters from the training cohort, the random forest algorithm was utilized to predict response employing the RandomForestClassifier() function from sklearn.ensemble and the area under the receiver operating characteristic curve score was computed.

1. Lindsley RC, et al. Prognostic Mutations in Myelodysplastic Syndrome after Stem-Cell Transplantation. *N Engl J Med*. 2017;376(6):536–547.

2. Lippert RA. Space-Efficient Whole Genome Comparisons with Burrows–Wheeler Transforms. *J Comput Biol*. 2005;12(4):407–415.

3. McKenna A, et al. The Genome Analysis Toolkit: A MapReduce framework for analyzing next-generation DNA sequencing data. *Genome Res*. 2010;20(9):1297–1303.

4. Wilm A, et al. LoFreq: a sequence-quality aware, ultra-sensitive variant caller for uncovering cell-population heterogeneity from high-throughput sequencing datasets. *Nucleic Acids Res*. 2012;40(22):11189–11201.

5. Wang K, Li M, Hakonarson H. ANNOVAR: functional annotation of genetic variants from high-throughput sequencing data. *Nucleic Acids Res*. 2010;38(16):e164–e164.

6. Jian X, Boerwinkle E, Liu X. In silico prediction of splice-altering single nucleotide variants in the human genome. *Nucleic Acids Res*. 2014;42(22):13534–13544.

7. The 1000 Genomes Project Consortium. A map of human genome variation from population-scale sequencing. *Nature*. 2010;467(7319):1061–1073.

8. Forbes SA, et al. COSMIC: mining complete cancer genomes in the Catalogue of Somatic Mutations in Cancer. *Nucleic Acids Res*. 2011;39(Database):D945–D950.

9. Akalin A, et al. Base-Pair Resolution DNA Methylation Sequencing Reveals Profoundly Divergent Epigenetic Landscapes in Acute Myeloid Leukemia. *PLoS Genet*. 2012.

10. Team RC. R: A language and environment for statistical computing. *R Found Stat Comput Vienna Austria*. 2021.

11. Dray S, Dufour A-B. The **ade4** Package: Implementing the Duality Diagram for Ecologists. *J Stat Softw*. 2007;22(4). https://doi.org/10.18637/jss.v022.i04.

12. Krueger F, et al. FelixKrueger/TrimGalore: v0.6.10 - add default decompression path. 2023. https://zenodo.org/record/7598955. Accessed August 4, 2023.

13. Langmead B, Salzberg SL. Fast gapped-read alignment with Bowtie 2. *Nat Methods*. 2012;9(4):357–359.

14. Krueger F, Andrews SR. Bismark: a flexible aligner and methylation caller for Bisulfite-Seq applications. *Bioinformatics*. 2011;27(11):1571–1572.

15. Park Y, et al. MethylSig: a whole genome DNA methylation analysis pipeline. *Bioinformatics*. 2014;30(17):2414–2422.

16. Goldfarb T, et al. NCBI RefSeq: reference sequence standards through 25 years of curation and annotation. *Nucleic Acids Res*. 2025;53(D1):D243–D257.

17. Subramanian S, et al. Genome-wide transcription factor–binding maps reveal cell-specific changes in the regulatory architecture of human HSPCs. *Blood*. 2023;142(17):1448–1462.

18. Wang Q, et al. Exploring Epigenomic Datasets by ChIPseeker. *Curr Protoc*. 2022;2(10). https://doi.org/10.1002/cpz1.585.

19. Kolde R. Pheatmap: pretty heatmaps. *R Package Version*. 2012;1(2):726.

20. *FastQC*. 2015.

21. Ewels P, et al. MultiQC: summarize analysis results for multiple tools and samples in a single report. *Bioinformatics*. 2016;32(19):3047–3048.

22. Dobin A, et al. STAR: ultrafast universal RNA-seq aligner. *Bioinformatics*. 2013;29(1):15–21.

23. Liao Y, Smyth GK, Shi W. featureCounts: an efficient general purpose program for assigning sequence reads to genomic features. *Bioinformatics*. 2014;30(7):923–930.

24. Love MI, Huber W, Anders S. Moderated estimation of fold change and dispersion for RNA-seq data with DESeq2. *Genome Biol*. 2014;15(12):550.

25. Greenberg P, et al. International Scoring System for Evaluating Prognosis in Myelodysplastic Syndromes. *Blood*. 1997;89(6):2079–2088.

26. Bernard E, et al. Molecular International Prognostic Scoring System for Myelodysplastic Syndromes. *NEJM Evid*. 2022;1(7). https://doi.org/10.1056/EVIDoa2200008.

27. Greenberg PL, et al. Revised International Prognostic Scoring System for Myelodysplastic Syndromes. *Blood*. 2012;120(12):2454–2465.

28. Khoury JD, et al. The 5th edition of the World Health Organization Classification of Haematolymphoid Tumours: Myeloid and Histiocytic/Dendritic Neoplasms. *Leukemia*. 2022;36(7):1703–1719.

29. Subramanian A, et al. Gene set enrichment analysis: A knowledge-based approach for interpreting genome-wide expression profiles. *Proc Natl Acad Sci*. 2005;102(43):15545–15550.

30. Durand NC, et al. Juicer Provides a One-Click System for Analyzing Loop-Resolution Hi-C Experiments. *Cell Syst*. 2016;3(1):95–98.

31. Telonis AG, et al. MIR retrotransposons link the epigenome and the transcriptome of coding genes in acute myeloid leukemia. *Nat Commun*. 2022;13(1):6524.

32. Szklarczyk D, et al. STRING v11: protein–protein association networks with increased coverage, supporting functional discovery in genome-wide experimental datasets. *Nucleic Acids Res*. 2019;47(D1):D607–D613.

33. Shannon P, et al. Cytoscape: A Software Environment for Integrated Models of Biomolecular Interaction Networks. *Genome Res*. 2003;13(11):2498–2504.

34. Kanehisa M. KEGG: Kyoto Encyclopedia of Genes and Genomes. *Nucleic Acids Res*. 2000;28(1):27–30.

35. Pedregosa F, et al. Scikit-learn: Machine Learning in Python. *J Mach Learn Res*. 2011;12(85):2825–2830.

SUPPLEMENTARY TABLES:

Supplemental Table 1: Cohort Characteristics. A table describing the patient information for samples used in the study.

Supplemental Table 2: Differentially expressed genes in MDS vs. Healthy. Related to Supplementary Figure 1. List of differentially expressed genes in MDS compared to healthy controls (absolute fold change ≥ 1.5, FDR ≤ 0.05). A positive log2 fold-change corresponds to increased gene expression in MDS patients.

Supplemental Table 3: Pathway analysis of differentially expressed genes and differentially methylated regions in MDS vs. Healthy. Related to Figure 1 and Supplementary Figure 1. Enriched pathways associated with differentially methylated regions between MDS and healthy controls, as well as enriched pathways identified through gene set enrichment analysis using RNA-seq data.

Supplemental Table 4: Quality control metrics for DNA methylation data. A table summarizing the number of CpG tiles captured at different coverage thresholds (coverage > 0, coverage ≥ 10 and coverage between 10 and 400).

Supplemental Table 5: Differentially methylated regions of MDS vs. Healthy. Related to Figure 1. List of differentially methylated regions in MDS compared to healthy controls (absolute methylation difference ≥ 25%, FDR ≤ 0.05).

Supplemental Table 6: Clinical and molecular characteristics of MDS DNA methylation clusters. Related to Figure 2. Significant molecular and clinical features of each MDS cluster, identified using Fisher's exact test

Supplemental Table 7: Differentially methylated regions of each Cluster vs. Healthy. Related to Figure 3 and Supplementary Figure 4. List of differentially methylated regions in each MDS cluster compared to healthy controls (absolute methylation difference ≥ 25%, FDR ≤ 0.05).

Supplemental Table 8: Pathway analysis of differentially expressed genes and differentially methylated regions in Cluster VI vs. Healthy. Related to Figure 3. Enriched pathways associated with differentially methylated regions in MDS Cluster VI compared to healthy control, as well as enriched pathways identified through gene set enrichment analysis using RNA-seq data.

Supplemental Table 9: Differentially expressed genes of Cluster VI vs. Healthy. Related to Figure 3. List of differentially expressed genes in MDS Cluster VI compared to healthy controls (absolute fold change ≥ 1.5, FDR ≤ 0.05). A positive log2 fold-change corresponds to increased gene expression in MDS Cluster VI.

Supplemental Table 10: **Differentially expressed genes of AZA responders vs. Non-responders.** Related to Figure 4. List of differentially expressed genes in AZA responders

compared to AZA non-responders (absolute fold change ≥ 1.5, FDR ≤ 0.05). A positive log2 fold-change corresponds to increased gene expression in AZA responders.

Supplemental Table 11: Pathway analysis of differentially expressed genes and differentially methylated regions in of AZA responders vs. Non-responders. Related to Figure 4. Enriched pathways associated with differentially methylated regions in AZA responders compared to non-responders, as well as enriched pathways identified through gene set enrichment analysis using RNA-seq data.

Supplemental Table 12: Differentially methylated regions of AZA responders vs. Non-responders. Related to Figure 4. List of differentially methylated regions in AZA responders compared to AZA non-responders (absolute methylation difference ≥ 25%, FDR ≤ 0.05).

Supplemental Table 13: Differentially methylated regions of AZA Complete responders vs. Non-responders. Related to Figure 4. List of differentially methylated regions in AZA complete responders compared to AZA non-responders (absolute methylation difference ≥ 25%, FDR ≤ 0.05).

Supplemental Table 14: Pathway analysis of differentially expressed genes and differentially methylated regions in of AZA Complete responders vs. Non-responders. Related to Figure 4. Enriched pathways associated with differentially

methylated regions in AZA complete responders compared to non-responders, as well as enriched pathways identified through gene set enrichment analysis using RNA-seq data.

Supplemental Table 15: Machine Learning models using DNAme data. Related to Figure 5. Input data matrix and train/test dataset used for machine learning modeling based on DNA methylation data.

Supplemental Table 16: Machine Learning models using DNAme, gene expression, mutation and clinical data. Related to Figure 5. Input data matrix and train/test dataset for machine learning modeling using DNA methylation, gene expression, mutation and clinical data.

SUPPLEMENTARY FIGURES:

Supplemental Figure 1:

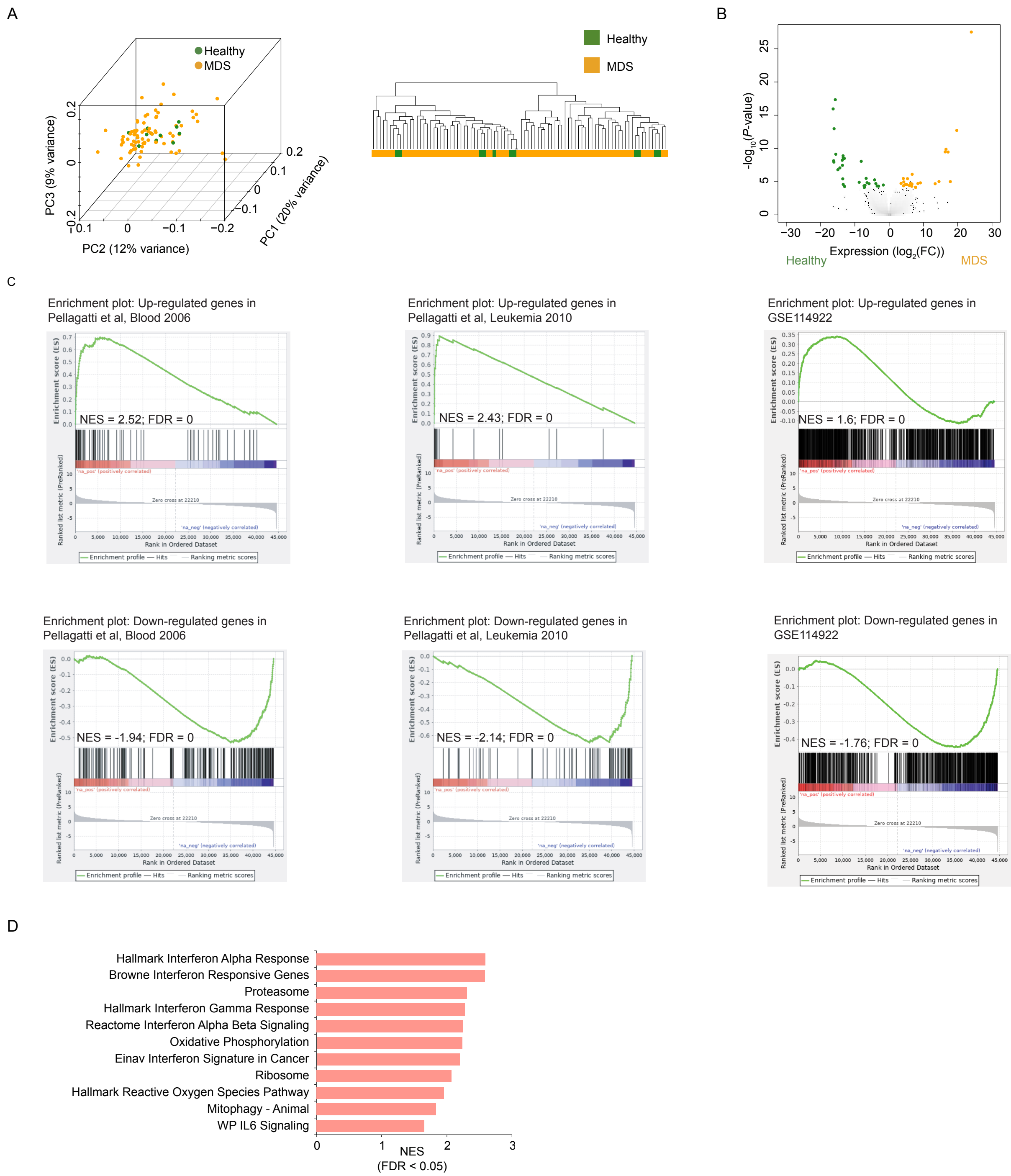

Supplemental Figure 1. Transcriptomic Profiling in MDS Patients. (**A**)**:** Correspondence analysis (*left*) and hierarchical clustering (*right*) of RNA-seq data, comparing healthy controls (green) and MDS patients (yellow). (**B**)**:** Volcano plot of the

$\log_2$fold-change (MDS/Healthy) gene expression versus the $-\log_{10}$(*P*-value). Downregulated and upregulated genes in MDS patients are shown in green and yellow, respectively (absolute fold change ≥ 1.5, FDR ≤ 0.05). (**C**)**:** Enrichment plots of DEGs from other published MDS cohorts* in our dataset. The normalized enrichment score (NES) and FDR scores are listed. (**D**)**:** Bar plot displaying the normalized enrichment score (NES) for pathways enriched in MDS compared with healthy controls. Pathways with increased activity are shown in red, while those with decreased activity are shown in blue (FDR ≤ 0.05).

*Pellagatti, Andrea, et al, Blood 2006, Pellagatti, Andrea, et al, Leukemia 2010, Pellagatti, Andrea, et al, Blood 2018

Supplemental Figure 2:

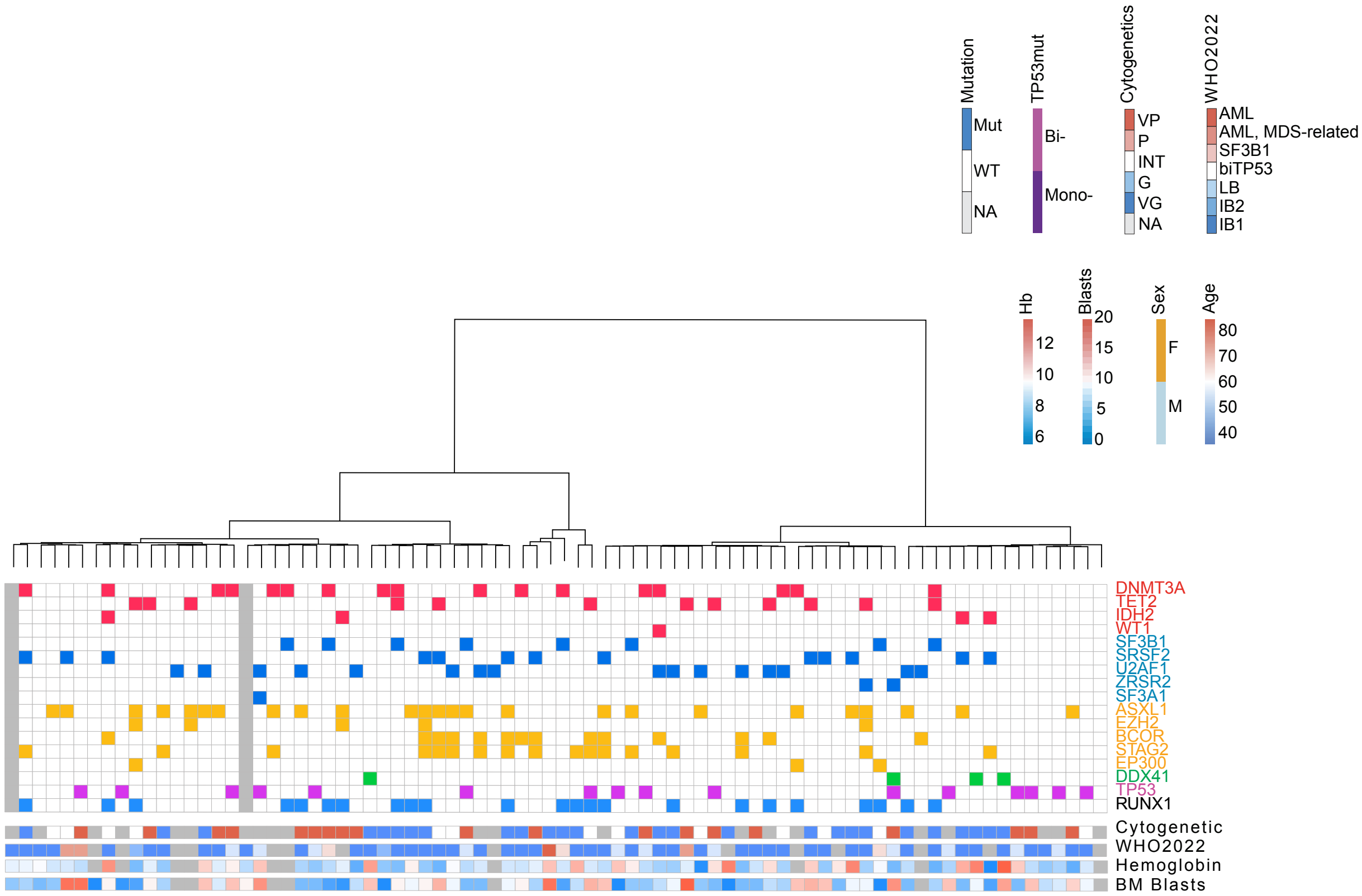

Supplemental Figure 2. Clustering of MDS Patients Based on Gene Expression Level. Heatmap and hierarchical clustering of MDS patients based on RNA-seq data, Corresponding mutation and clinical annotations are displayed below, with patients harboring gene mutations color-coded. Cytogenetic risk: VP, very poor; P, poor; INT, Intermediate; G, good; VG, very good; NA, not available. WHO 2022: IB1, MDS with increased blasts-1; IB2, MDS with increased blasts-2; LB, MDS with low blasts.

Supplemental Figure 3:

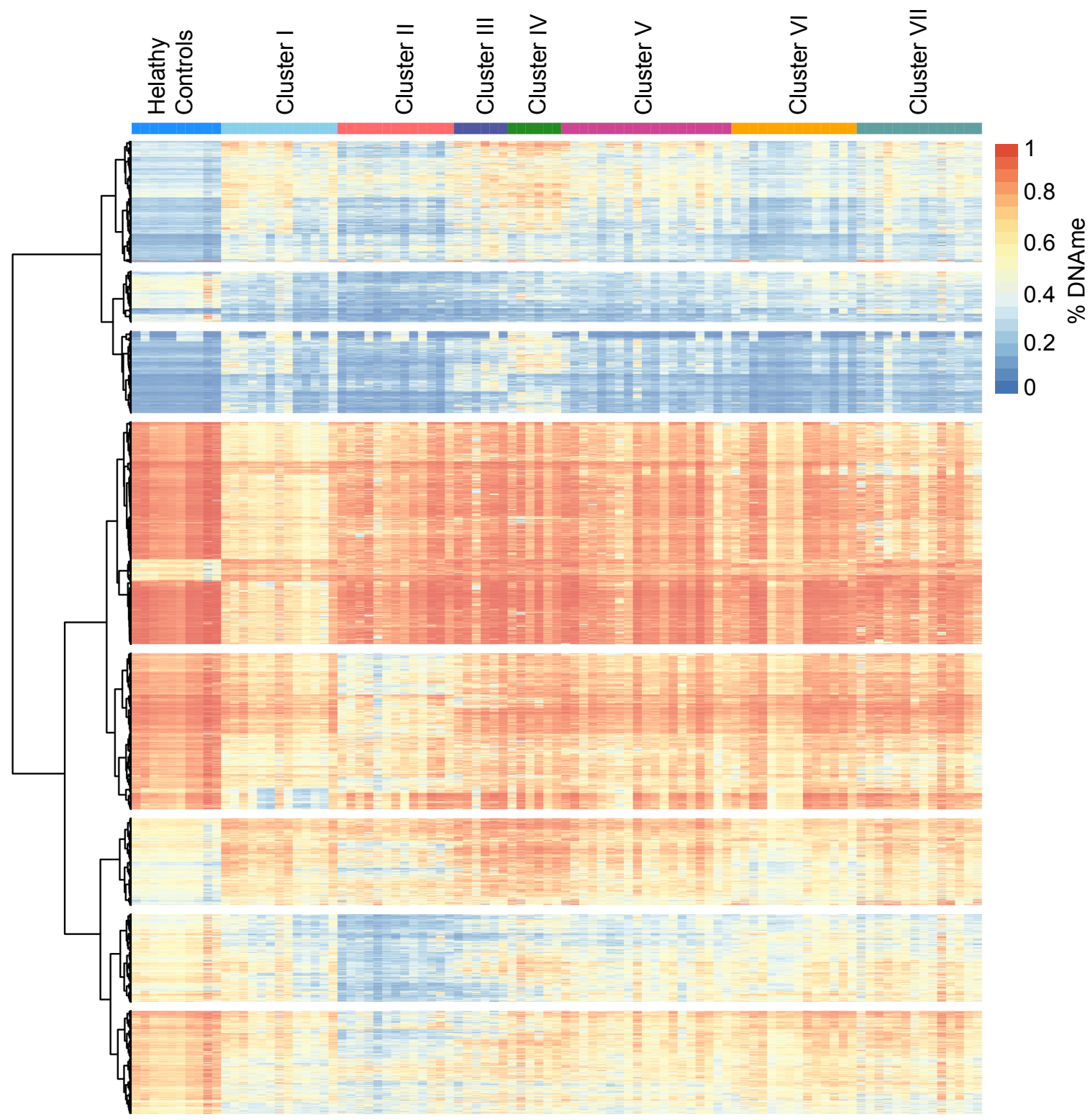

Supplemental Figure 3. Heatmap of DNA Methylation Levels in MDS patient clusters. Heatmap illustrating DNA methylation levels across seven MDS patient clusters and healthy controls. DNAme, DNA methylation.

Supplemental Figure 4:

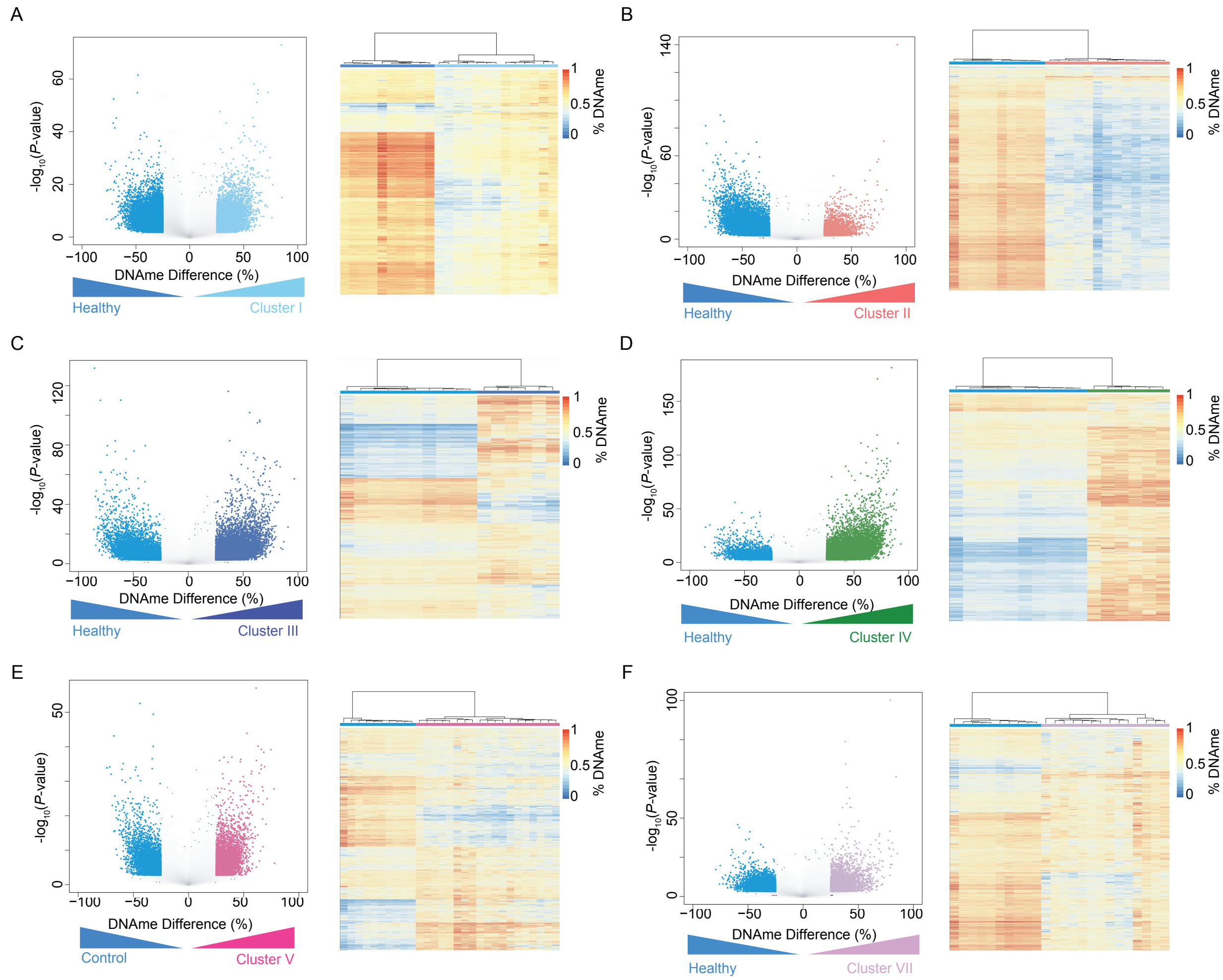

Supplemental Figure 4. Differential Methylation in MDS Clusters Compared to Healthy Controls. **A-F:** Volcano plots (*left*) illustrating methylation differences versus the -$\log_{10}$(*P*-value) between healthy controls and each MDS cluster (absolute methylation difference ≥ 25%, FDR ≤ 0.05). Corresponding heatmaps (*right*) display methylation levels of differentially methylated regions in healthy controls (blue bar) and MDS clusters: I (light blue), II (red), III (dark blue), IV (green), V (dark pink), and VII (purple). DNAme, DNA methylation.

Supplemental Figure 5:

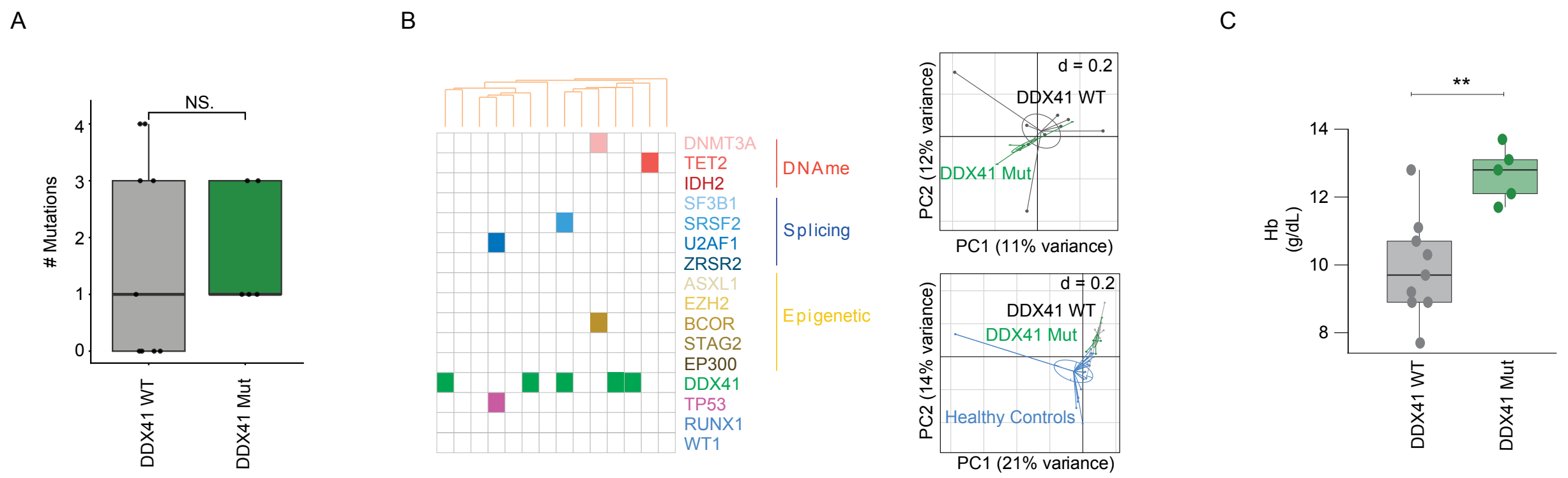

Supplemental Figure 5. Epigenetic and Molecular Characterization of *DDX41*-mutant and *DDX41*-wild-type cases in Cluster VI. (**A**)**:** Box plot illustrating the number of mutations per patient across *DDX41*-mutant (green) and *DDX41*-wild-type (grey) cases in Cluster VI. The bounds of the boxes represent the 25th and 75th percentiles, the horizontal line within the box indicates the median, and the whiskers extend to the minimum and maximum values. Individual outliers are represented by dots. *P* value was calculated using the Wilcoxon rank sum test. NS, non-significant (*P* > 0.05). (**B**)**:** Hierarchical clustering of MDS patients within Cluster VI, corresponding mutations are displayed below, with specific gene mutations indicated by color coding (*left*). Principal Components Analysis (*right*) of *DDX41*-mutant (green) and *DDX41*-wild-type (grey) patients within Cluster VI, visualized without (*upper*) and with (*bottom*) healthy controls (blue). (**C**)**:** Box plot showing the distribution of hemoglobin (Hb) levels in *DDX41*-wild-type (grey) and *DDX41*-mutant (green) patients within Cluster VI. The bounds of the boxes represent the 25th and 75th percentiles, the horizontal line within the box indicates the median, and the whiskers extend to the minimum and maximum values. Individual outliers are represented by dots. *P* values were calculated using the Wilcoxon rank sum test. **, *P* ≤ 0.01.

Supplemental Figure 6:

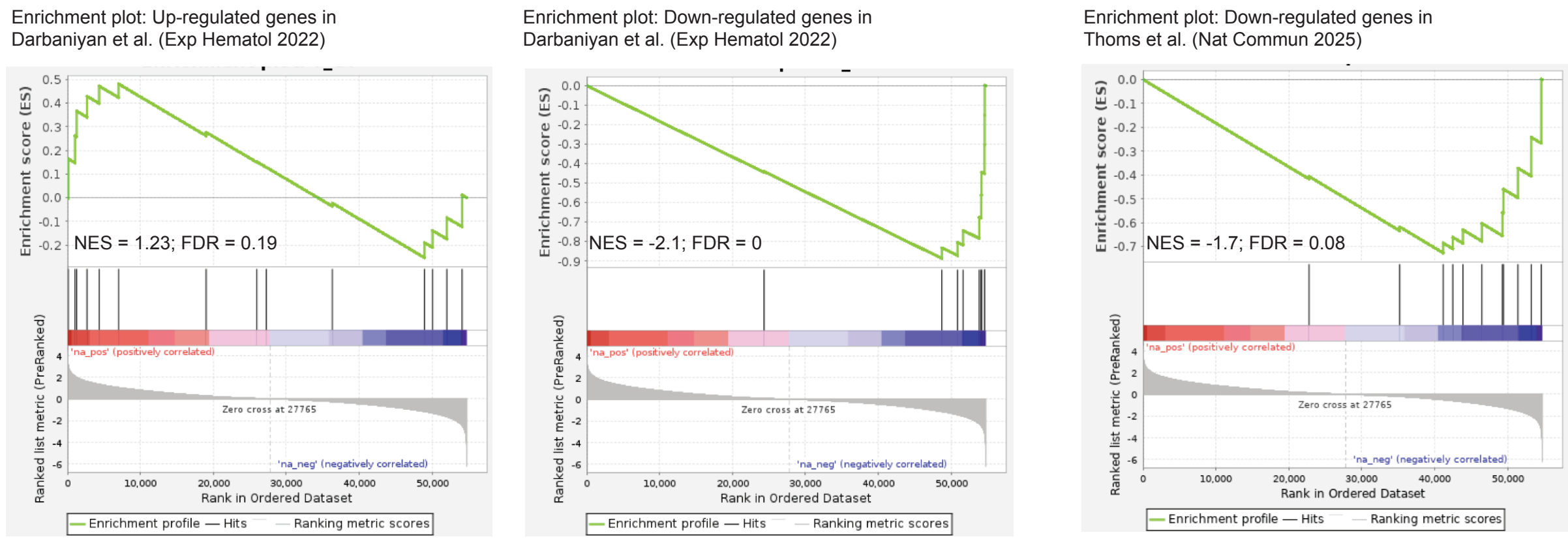

Supplemental Figure 6. Enrichment plots of differentially expressed genes (DEGs) reported in previous publications* and evaluated in our dataset. The normalized enrichment score (NES) and FDR are shown.

*Darbaniyan, Faezeh, et al, Experimental Hematology 2022, Thoms, Julie AI, et al, Nature Communications 2025

Supplemental Figure 7:

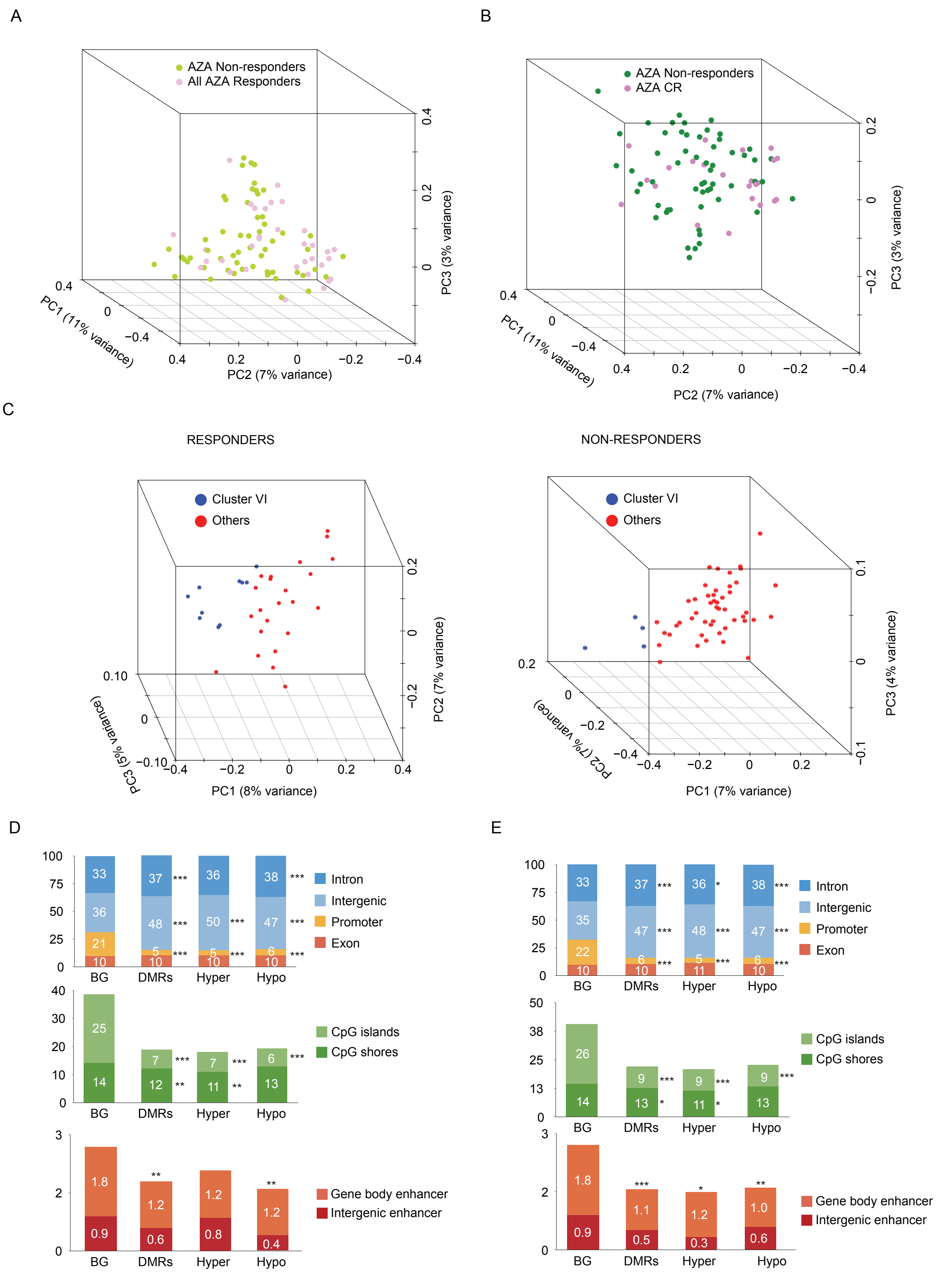

Supplemental Figure 7. Characterizing Methylation Differences and Pathway Enrichment in AZA Non-Responders and Responders/Complete Responders. (**A**)**:** Principal Component Analysis of ERRBS data comparing AZA non-responders (green) and all responders (light purple). (**B**)**:** Principal Component Analysis of ERRBS data comparing AZA non-responders (dark green) and complete responders (dark purple). (**C**)**:** Principal Component Analysis of ERRBS data comparing MDS Cluster VI responders (blue) with all other responders (red) (left), and MDS Cluster VI non-responders (blue) with all other non-responders (red) (right). (**D**)**:** Stacked bar plot showing the distribution of differentially methylated regions between AZA non-responders and responders across genomic regions, CpG islands, and enhancers. *P* values were calculated using a binomial test. *, $P \leq 0.05$; ***, $P \leq 0.001$. (**E**)**:** Stacked bar plot showing the distribution of differentially methylated regions between AZA non-responders and complete responders across genomic regions, CpG islands, and enhancers. *P* values were calculated using a binomial test. *, $P \leq 0.05$; ***, $P \leq 0.001$.

Supplemental Figure 8:

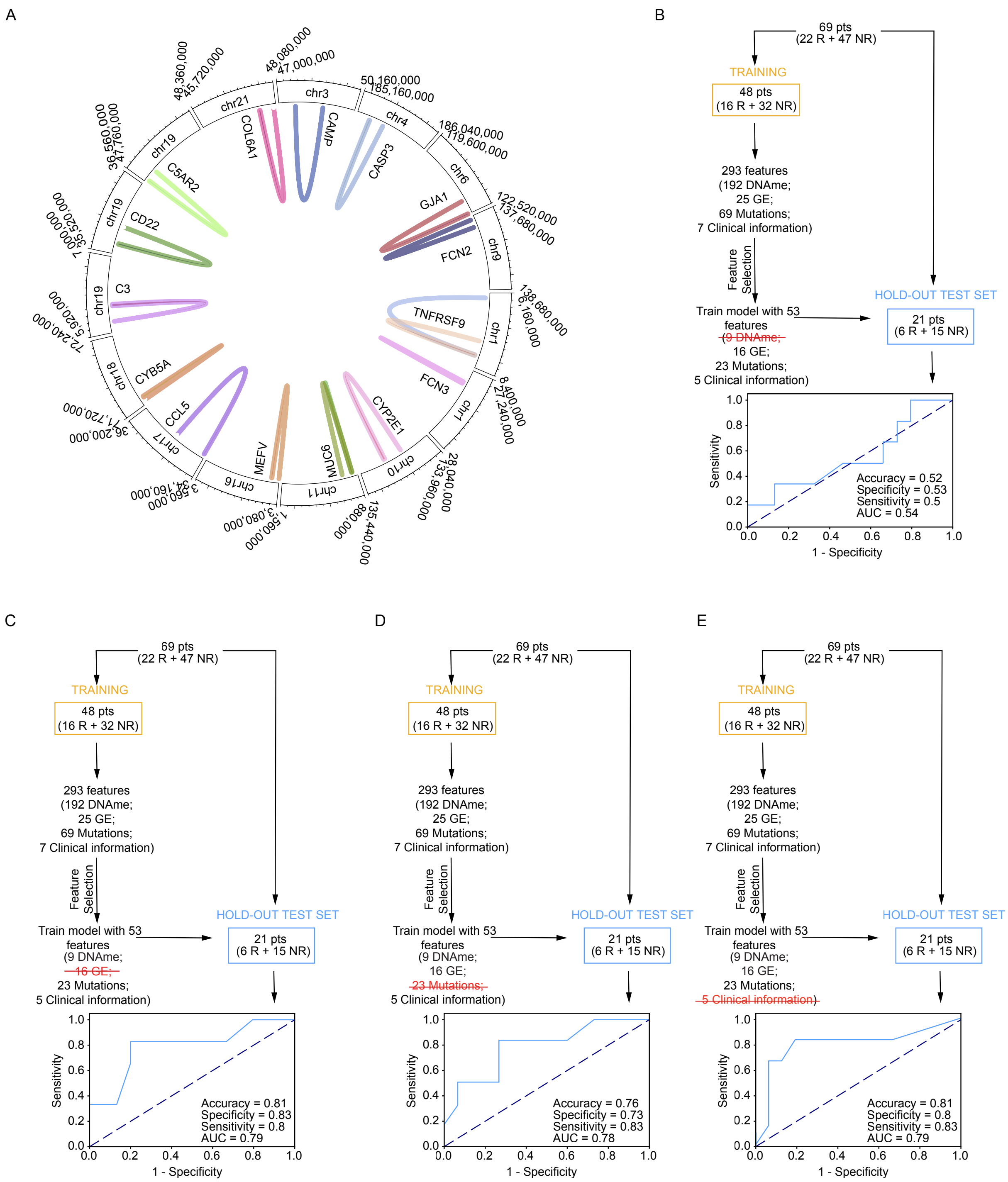

Supplemental Figure 8. Genes Correlated with DMRs in the Same TAD Regions and Machine Learning Performance with Different Input Features. (**A**): Circos plot illustrating the genomic coordinates of differentially methylated regions and their correlated genes (|Pearson correlation| > 0.7) within the same topologically associating

domain regions. (**B**)**:** Workflow of machine learning predictor (from Figure 5D) for distinguishing AZA responders from non-responders after removing DNA Methylation features. (**C**)**:** Workflow of machine learning predictor (from Figure 5D) after removing gene expression features. (**D**)**:** Workflow of machine learning predictor (from Figure 5D) after removing mutation features. (**E**)**:** Workflow of machine learning predictor (from Figure 5D) after removing clinical features.
